# Antimicrobial evaluation of *Cardiospermum halicacabum* L. leaf fractions and its usage in active bioactive film formation for shelf-life enhancement in fresh-cut fruits

**DOI:** 10.3389/fmicb.2026.1812196

**Published:** 2026-04-01

**Authors:** Uma Venkatesan, Rajiniraja Muniyan

**Affiliations:** School of Bio Sciences and Technology, Vellore Institute of Technology, Vellore, India

**Keywords:** antimicrobial activity, cytotoxicity, fractionation, molecular dynamics, preservation

## Abstract

Food wrapping plays a critical role in maintaining the superior quality, protection, and preservation of food commodities by preventing contamination and spoilage. The current study investigated the biological potential, chromatographic profiling, molecular docking, and simulation studies of *Cardiospermum halicacabum* L. leaf fractions. Furthermore, the bioactive fraction of *C. halicacabum* was incorporated with chitosan and pectin matrices (Chi/Pec/CF) to develop an innovative antioxidative and antibacterial edible film for packaging fresh-cut fruit. Antioxidant and antimicrobial activities were estimated using different solvent extracts (petroleum ether, chloroform, and ethanol). Among these, the ethanol extract exhibited the highest free radical scavenging activity and the strongest antimicrobial activity. Furthermore, bioactivity-guided fractionation with antimicrobial activity was performed using column chromatography. The bioactive fraction was subjected to GC–MS analysis, identifying 27 phytocomponents, which were subsequently evaluated by docking analysis against DNA gyrase B from *S. aureus* and dihydrofolate reductase from *E. coli.* The results indicated that three compounds exhibited higher binding affinities than the reference compounds. The docking scores of Hit 1 (4′-pentylbicyclohexyl-4-carboxamide) were −9.57 and −16.33 kcal/mol, Hit 2 (2-formyl-9-[beta-d-ribofuranosyl] hypoxanthine) were −10.95 and −15.00 kcal/mol, and Hit 3 (2-hydroxymethyl-9-[beta-d-ribofuranosyl] hypoxanthine) were −11.18 and −14.80 kcal/mol against the respective target proteins. Moreover, the overall bioactive fraction was used to develop the Chi/Pec/CF film as a packaging material, and it was evaluated for extending the shelf life of fresh apple slices stored for 6 days at 4 °C. Overall, the results highlight the potential of these composite films as promising food packaging materials owing to their antimicrobial, antioxidative, and biodegradable properties.

## 1 Introduction

Foodborne pathogenic bacteria are responsible for crucial illnesses affecting both humans and animals. They represent a major public concern in both industrialized and developing nations (Mendez et al., 2012). Approximately 600 million people worldwide suffer from foodborne infections and diarrheal illnesses, resulting in 420,000 fatalities per year (Das et al., 2024). Conventional food preservation methods require the use of several physical and chemical techniques to inhibit bacterial growth. Packaging constituents may be derived from biological resources, including decomposable and sustainable biopolymers, as a substitute for conventional synthetic substances that may impact public health. Traditional medicines (TMs) are extensively utilized in many countries, such as India, Pakistan, China, Korea, Thailand, Japan, and Sri Lanka. For a long time, medicinal plants have been used by people to treat a variety of illnesses and endure either directly or indirectly as additives nowadays (Homayounpour et al., 2021; Salem et al., 2021). Natural food additives with fewer side effects are recommended because synthetic ones might alter the body’s cellular structure and have various negative impacts. When natural resources replace synthetic antimicrobial drugs, foodborne pathogens are less likely to develop antibiotic resistance. Research into finding natural antioxidants for their therapeutic benefits and substituting synthetic antioxidants that are known to have potential carcinogenic implications has significantly increased recently. Plant-based extracts can be utilized as bio-preservatives to reduce the use of synthetics because they have antibacterial and antioxidant properties. Conversely, natural components, also referred to as GRAS antioxidants, are safe, stable during processing, and extend durability. They are effective at low concentrations and are unlikely to adversely affect sensory qualities. Moreover, compared to synthetic antioxidants, natural ones are nontoxic at high concentrations (Shi et al., 2024; Venkatesan and Muniyan, 2024).

The genus *Cardiospermum halicacabum* L., which belongs to the *Sapindaceae* family, contains about 17 species of vines and herbs that grow in tropical climates throughout the world. Numerous studies and coastal populations’ traditional knowledge indicate that medicinal plants have potent anti-inflammatory, antiviral, antibacterial, anticancer, anti-diabetic, antifungal, and insecticidal characteristics (Gaziano et al., 2018). Balloon vine, or *C. halicacabum*, is a climbing plant that is frequently employed in herbal medicine to treat a variety of ailments, including oxidative stress-related illnesses, microbial infections, and inflammation (Roy et al., 2021). Because of its affordability, the *A. cepa* assay was used in this investigation to screen for cytotoxicity, and the method produced a significant amount of data with simple culture techniques and was free from ethical issues related to implementing animals in toxicity assessments (Alabi et al., 2022). Due to their antioxidant properties, phenolic and flavonoid components are crucial for scavenging free radicals while decreasing oxidative stress, two factors related to the development of chronic illnesses like cancer and cardiovascular disorders. Similarly, plant-derived antimicrobial substances reveal potency against both Gram-positive and Gram-negative bacteria, providing a natural substitute for synthetic preservatives. Moreover, specific cytotoxic phytoconstituents establish importance in anticancer research and require further investigation into their possible effects and safety (Rice-Evans et al., 1997; Fenech, 2000).

The functions of the binding sites for DNA Gyrase B (GyrB) from *Staphylococcus aureus* and dihydrofolate reductase (DHFR) from *Escherichia coli* have been reported in earlier studies. DNA gyrase signifies possible targets for antimicrobial drugs. Within the two subunits of DNA gyrase, GyrA is responsible for double-strand DNA cleavage, whereas GyrB is involved in ATPase activity. The mode of action of antibiotics that inhibit DNA gyrase acts on unique domains within these two subunits. GyrB, a subunit of the bacterial enzyme that generates the tetramer along with GyrA subunits, is essential for DNA supercoiling through ATP binding and hydrolysis. This activity leads to the translocation of DNA strands during transcription and replication. It comprises ATP binding sites and a core with the enzyme’s catalytic functions. On the other hand, the DHFR protein is a metabolic enzyme in the folic acid pathway that facilitates the transformation of dihydrofolate into tetrahydrofolate, and it is crucial for cell proliferation via thymidine synthesis. It is a key gene product and represents an important target for the elimination of antibiotic-resistant bacteria (Erarslan and Yuka, 2024).

Moreover, bioactive packaging is an innovative strategy to sustain the nutritious integrity of fresh produce by protecting it from microbial contaminants and preventing deterioration caused by biological and chemical alterations. Employing natural polymers, including lipids, proteins, polysaccharides, and their blends, has been identified as the foremost potential and environmentally sustainable method. The blending of polymers that degrade easily with natural components to generate edible films and coatings has been examined as an effective strategy for the food industry. Chitosan is a natural polymer derived from chitin and obtained from the shells of marine crustaceans. It can be chemically or enzymatically modified for many applications and is characterized by its biodegradable, biocompatible, and non-toxic attributes, which are utilized in numerous fields, including medical, cosmetics, agriculture, textiles, paper, and food industries. Pectin is another comprehensively investigated polysaccharide among many polymers produced from plants due to its non-toxic nature, affordability, availability, lower cost, and biodegradability. The integration of polymers with naturally occurring active components allows the production of usable films with higher quality and technological capabilities (Subramani and Manian, 2024; Venkatesan and Muniyan, 2024).

Therefore, the present study aimed to investigate a partially purified ethanolic extract of *C. halicacabum* using both *in silico* and *in vitro* approaches against foodborne microorganisms. The research evaluated antioxidant, antimicrobial, and cytotoxicity activities along with docking and simulation analyses targeting GyrB and DHFR proteins. Furthermore, the bioactive fraction was incorporated into a chitosan-pectin composite edible film to assess its potential as a natural bio-preservative. The developed film was applied to fresh-cut apple slices to estimate its effectiveness in prolonging their shelf life for 6 days during storage at 4 °C.

### 1.1 Sample acquisition and extraction using Soxhlet apparatus

The field of research in the current study was performed in compliance with the relevant institutional norms and regulations. No endangered or protected species were involved, and the work adhered to both institutional and national guidelines. The fresh plant leaves of *C. halicacabum* were obtained from the VIT University campus located in Vellore, Tamil Nadu, India. The collected leaves were taxonomically identified by Dr. C. Rajasekaran from the Plant Biotechnology Department at VIT University, and the voucher specimen no. (*Cardiospermum halicacabum* - VITOD01-216).

### 1.2 Soxhlet extraction

Twenty-five grams of dried *C. halicacabum* leaf powder were subjected to 300 mL of three various solvents, such as petroleum ether, chloroform, and ethanol, via the Soxhlet apparatus based on their increasing polarity. After 8 h, each solvent was extracted separately based on its boiling point, with petroleum ether for non-polar compounds (around 40–60 °C), chloroform for moderately polar compounds (61.2 °C), and ethanol for polar substances (above 78.5 °C). A rotary evaporator was used to remove the solvent after each extraction, and the extracts were dried, and their weights (yields) determined. For further bioassay investigations, phytochemicals with the dried extracts were kept at 4 °C in amber vials (Bontzolis et al., 2024).

### 1.3 Estimation of free radical scavenging activity

#### 1.3.1 DPPH (2,2-diphenyl-1-picrylhydrazyl) assay

To estimate the different solvent extracts to measure the free radical scavenging activity, the DPPH assay was conducted using previously published methods with minor modifications. A standard stock solution of DPPH (0.1 mM) was prepared in methanol, and plant extracts with different concentrations (50–250 μg/mL) were added to a test tube containing 3.9 mL of the DPPH solution. After allowing the mixture to remain at ambient temperature in the dark for half an hour, a UV–Vis spectrophotometer was used to record the absorbance at 517 nm (Venkatesan and Muniyan, 2025; Asati et al., 2021). Using the following formula, the DPPH radical scavenging activity was expressed as a percentage:


$$\%\text{of antioxidant activity}=\frac{\begin{array}{l} \text{absorbance of control}-\\ \text{absorbance of sample} \end{array}}{\text{absorbance of control}}\times 100$$

### 1.4 Estimation of antibacterial activity

The antibacterial effectiveness of selected plants was determined using the well diffusion technique. The two bacterial foodborne pathogens used as test pathogens were *Staphylococcus aureus—*MTCC1144 and *Escherichia coli—*MTCC452.

#### 1.4.1 Well diffusion method

The previously reported well diffusion method was used to determine the antibacterial properties of the *C. halicacabum* various extracts, with slight modifications. Four wells (8 mm) were made in the agar medium using a sterile cork borer, and 30 μL of the various concentrations (10–25 mg/mL) of the extracts and the reference drug tetracycline (1 mg/mL) were added. For the extracts and reference drugs, three separate instances of the experiment were conducted, and the antibacterial plates were maintained for 24 h at 37 °C. The mean values were obtained by measuring the diameter of the growth–inhibition zone (Venkatesan and Muniyan, 2025).

### 1.5 Cytotoxicity assay

#### 1.5.1 Evaluation of *Allium cepa* root tip test

The *A. cepa* root tip assay was used to evaluate the cytotoxicity of the bioactive extract with minor modifications. To prevent damage to the root primordia, healthy *A. cepa* bulbs were carefully selected, and their dry outer scales were removed. The bulbs were then submerged in distilled water to promote root development until they reached 2–3 cm in length. The roots were subsequently exposed to plant extract solutions for 48 h at different concentrations of 25, 50, 75, and 100 μg/mL, with distilled water serving as the negative control. After exposure, the roots were rinsed with distilled water and preserved for 24 h in Carnoy’s fixative solution (3:1 ethanol: acetic acid). After hydrolysis for 5 min at 60 °C in 1 N HCl, the root tips were treated with 2% acetocarmine and then squeezed onto glass slides. To evaluate the extract’s impact on cell division, these slides were examined under a light microscope for chromosomal aberrations (Alias et al., 2023).

### 1.6 Chromatographic techniques

#### 1.6.1 Thin-layer chromatography

TLC was used to separate the phytocomponents in the presence of ethanolic extract of *C. halicacabum.* Different solvents with varying polarities were employed, such as benzene: ethyl acetate (4:1), petroleum ether: ethyl acetate (4:1), hexane: ethyl acetate (4:1), and toluene: ethyl acetate (4:1). From the comparison of all solvents, a toluene: ethyl acetate (4:1) ratio was estimated to be optimal for chromatography. Furthermore, 2 μL of the extract was spotted on TLC plates precoated with silica gel and allowed to dry immediately. The spotted plates were then viewed under UV light at 366 nm, and Rf values were calculated to identify the bioactive constituents using the formula (Chaqroune et al., 2021).


$$\begin{array}{l} \mathrm{Rf}\;\text{value}=\text{Distance travelled}\;\mathrm{by}\;\text{solute}/\\ \text{Distance travelled}\;\mathrm{by}\;\text{solvent} \end{array}$$

### 1.7 Column chromatography

The bioactive ethanolic extract was subjected to column chromatography for the separation of active compounds, with minor modifications, using toluene and ethyl acetate as eluting solvents. The eluted fractions were pooled based on similar results from TLC, and the fractions were submitted for antimicrobial screening (Che Sulaiman et al., 2017).

#### 1.7.1 Bioactivity-guided antimicrobial activity

The fractions were further examined using the agar well diffusion method against foodborne pathogens, namely *S. aureus* and *E. coli* (Mir et al., 2020). Following this, the analysis of the bioactive fraction was carried out using GC–MS.

### 1.8 Gas chromatography–mass spectrometry study

The bioactive fraction was processed for the detection of phytocomponents utilizing the GC–MS technique following the protocol (Venkatesan and Muniyan, 2025). Compound identification was performed by comparing the obtained mass spectra with those available in the NIST and Wiley spectral libraries integrated within the GC–MS software. The exported dataset included retention time and peak area information; however, detailed similarity index (match factor) values were not available. No confirmation using authentic standards, retention index comparison, or compound isolation was conducted. Therefore, compound assignments should be considered tentative.

### 1.9 Molecular docking studies

The docking analysis was performed using the tentatively identified compounds based on GC–MS library matching. The molecular structures of our protein targets, *S. aureus* DNA Gyrase B (GyrB; PDB ID: 3U2D) and *E. coli* dihydrofolate reductase (DHFR; PDB ID: 2ANQ), were utilized (Erarslan and Yuka, 2024). The ligand molecules were obtained in Structure-Data Files (SDF) format from PubChem^1^ and subsequently modified to PDB format with the Open Babel 3.3.1 program (O’Boyle et al., 2011). The protein structure was prepared using AutoDock 4.2 (Morris et al., 2009). All non-protein atoms, such as crystallographic water molecules, were removed throughout the processing method. The structure was prepared by adding polar hydrogens and assigning Kollman charges. The ligand molecules corresponding to Reference 1 (CID 9543473) and Reference 2 (CID 54759160) were acquired from the PubChem database and subjected to docking preparation using AutoDock 4.2. The prepared ligands were subsequently converted to the pdbqt format. The curated ligand library was then employed for virtual screening with AutoDock Vina 1.1.2 (Jaghoori et al., 2016). The molecular docking analysis was carried out using AutoDock Vina 1.1.2 under the labeled parameters to ensure reproducibility. For Reference compound 1, the grid box was centered at x = 29.540 Å, y = 43.195 Å, and z = 15.094 Å, with dimensions of 72 × 118 × 126 points and a grid spacing of 0.375 Å, encompassing the entire active site region. Similarly, for Reference compound 2, the grid box was centered at x = 26.517 Å, y = 44.721 Å, and z = 14.583 Å, with dimensions of 106 × 126 × 102 points and a grid spacing of 0.375 Å, fully containing the respective binding pocket. The docking parameters were set to exhaustiveness = 8, number of binding modes = 10, and energy range = 4 kcal/mol, while all other parameters were maintained at their default AutoDock Vina settings to ensure consistency across all runs.

The compounds that performed superiorly with AutoDock Vina were subsequently docked with the AutoDock tool using 100 iterations and the Lamarckian algorithm for both proteins. One protein with one ligand was handled at this stage with the same grid parameters as mentioned in the previous step. The interactions between proteins and ligands, including hydrogen bonds, hydrophobic contacts, and 2D/3D binding modes, were examined and visualized using Discovery Studio Visualizer (DSV).^2^

#### 1.9.1 Docking validation

The reliability of the docking methodology was evaluated by a redocking analysis of the co-crystallized ligand. The native ligand was removed from the active site and subsequently re-docked into the binding pocket using the same grid parameters and docking conditions applied to the test compounds. The redocked pose successfully reproduced the experimentally observed binding conformation, yielding a root-mean-square deviation (RMSD) value within an RMSD limit of 2.0 Å. Therefore, the results confirm the accuracy and validity of the docking protocol employed in the current study.

### 1.10 Molecular dynamic simulation studies

The stability of identified hits, references, and docked complexes was evaluated with GROMACS version 2023.1 software. The protein topology was prepared using pdb2gmx and the CHARMM27 force field, followed by solvation in a triclinic box with TIP3P water. Ligand topologies were obtained from the SwissParam server (Zoete et al., 2011). After neutralization with sodium ions, the protein–ligand complexes were solvated and subjected to energy minimization for 50,000 steps, followed by system equilibration. NVT and NPT ensembles were employed at 300 K and 1 bar for 100 ps each, with positional constraints applied. Ultimately, 200 ns molecular dynamics simulations were conducted for the references and protein–ligand complexes, and root-mean-square deviation (RMSD), root-mean-square fluctuations (RMSF), radius of gyration (Rg), solvent accessible surface area (SASA), and hydrogen bond interactions were computed and illustrated utilizing Xmgrace^3^ and VMD software (Venkatesan and Muniyan, 2025).

### 1.11 Statistical analysis

All statistical analyses were conducted with GraphPad Prism 8, and the results were expressed as mean ± SD (*n* = 3). Two-way ANOVA was used to evaluate the data, with *p* < 0.05 considered significant.

### 1.12 Preparation of bio-composite edible films

The edible film was produced in accordance with the methodology with a few modifications (Chaichi et al., 2017; Shivangi et al., 2021). The chitosan solution was obtained by dissolving 1% (v/v) acetic acid along with 1% (w/v) chitosan polymer and stirring in a magnetic stirrer for 24 h. Thereafter, glycerol (15% w/w, relative to polymer weight) was added to the chitosan solution. Subsequently, 4.5% of the extract was added to the polymer solution and stirred for 30 min, while the solution without extract was prepared as the control. After that, the mixture was combined with a pectin solution (1%, w/v) and continuously stirred to attain a final ratio of chitosan and pectin of 1:1 (w/w). The resulting film-forming solutions were cast onto petri plates and dried. After 48 h, the control and extract-loaded films were carefully peeled off and subjected to further characterization. The films produced were coded as Chitosan (Chi), Chitosan with active fraction (Chi/CF), Pectin (Pec), Pectin with active fraction (Pec/CF), Chitosan and Pectin (Chi/Pec), and Chitosan and Pectin with active fraction (Chi/Pec/CF). The edible film samples were analyzed utilizing a Shimadzu, Inc. UV-1800-type UV–visible spectrophotometer. A small portion of the edible films was analyzed for the light transmittance spectrum in the wavelength range of 200–700 nm (Sun et al., 2017; Subramani and Manian, 2024).

### 1.13 Characterization of edible films

#### 1.13.1 Morphological analysis of edible films with thickness, density and optical properties

The films were assessed at magnifications of 10×, 40×, and 100 × with an optical microscope. A UV spectrophotometer was used to ascertain the opacity values of the films, while a screw gauge was utilized to measure the films’ dimensions. The films were modified to possess uniform length and breadth in a rectangular format. The film densities were determined by utilizing the films’ size and mass. The film capacity was calculated using the surface area and the thickness of the layers of the film. The transparent cuvette containing the finely cut film was employed to assess the OD (optical density) at a 600 nm wavelength. The empty glass cuvette functioned as a blank (Subramani and Manian, 2024). The formula was utilized to determine the opacity values.


$$\text{Opacity}\;(\mathrm{mm}-1)=\frac{\text{absorbance}\;\mathrm{at}\;600\;(\mathrm{nm})}{\text{thickness}\;(\mathrm{mm})}$$

#### 1.13.2 Moisture content

Each film sample had an initial weight and was shriveled at 100 °C to obtain a dry weight. Then, the moisture content of the film sample (Subramani and Manian, 2024) was calculated with the following equation:


%moisture ratio=(initial mass−dried mass)/(initial mass)×100

#### 1.13.3 Solubility

To assess the solubility of films in water, each film was weighed and dipped in 5 mL of water separately and allowed to remain for 24 h. At the end of this time, each film sample was dried and weighed, and the solubility of percentages (Subramani and Manian, 2024) was estimated using the following equation:


%solubility=(initial mass−final mass)/(initial mass)×100

#### 1.13.4 Swelling degree analysis

The swelling ratio experiment was executed by modifying the methodology from the literature. The pectin films were maintained at ambient temperature, weighed, and immersed in deionized distilled water for wetting. Then, the excess amount of water was absorbed from the film using filter paper, and the wet film was promptly measured (Karkar et al., 2023; Adımcılar et al., 2025). Consequently, the swelling was determined using the following equation:


%swelling ratio=(wetmass to dried mass)/(dried mass)×100

#### 1.13.5 Water vapor permeation test

Each film sample was analyzed according to the existing protocol with a few modifications. Each film sample was initially weighed and then placed in test tubes containing 2 g of anhydrous CaCl₂, ensuring that they did not come into contact with CaCl₂. The film samples were kept in a moisture-free environment for 24 h and finally weighed. The rise in the density of films and water vapor permeability (Shivangi et al., 2021) was calculated using the following equation:

$\mathrm{WVP}=\frac{w}{t}$ $\times \frac{\delta }{A\times △P}$

where *w/t* is the slope of the weight loss (g) x time (h) curve, *δ* is the initial film thickness (mm), *A* is the surface area, and *ΔP* is the difference in vapor pressure between the inside and outside of the flask (0.7 kPa).

### 1.14 Structural analysis of edible films

#### 1.14.1 FTIR, XRD, NMR, and SEM analysis

The films were examined using Fourier Transform Infrared Spectroscopy (FTIR) spectra acquired in attenuated total reflectance mode with the Jasco 6,800 spectrometer (Jasco Inc., Japan), encompassing a spectrum of wave numbers from 500 to 4,000 cm^−1^. Both the functional characteristics in each sample and the associations among the film components were assessed. The X-ray diffractometer (XRD; Bruker, Inc., AXS D8 Advancement, Karlsruhe, Germany) was used to obtain an XRD image of the film sample at a voltage of 40 mA and 40 volts. Ni-filtered radiation from Cu K was utilized for that purpose. The technique of nuclear magnetic resonance spectrophotometry was applied for hydrogen nuclear magnetic resonance (1H NMR) determination with a Bruker Ascend 850 MHz spectrophotometer (Billerica, MA, United States), and carbon nuclear magnetic resonance (^13^C NMR) was obtained using the Bruker Advance 400 MHz FT-NMR spectrophotometer. To enhance the electron conductivity, film samples were split into small portions and sputtered with gold. The surface of each film was evaluated, including both morphology and microstructure of these films, utilizing the Quanta FEG 250 scanning electron microscope (SEM) at a 5 kV accelerating voltage. With supporting information, energy-dispersive X-ray (EDX) was analyzed for the presence of elements within these films (Wang et al., 2024; El Habbasha et al., 2025).

### 1.15 Thermal properties

Thermal strength assessments of the produced films were subjected to thermogravimetric analysis with Q50 V20.1 in a thermogravimetric analyzer (Newcastle, DE, United States). Ten mg of composite films were analyzed, and the samples were heated from 30 to 800 °C at a consistent rate of 20 °C per minute in a nitrogen (N₂) atmosphere (Akalin et al., 2022).

### 1.16 Antioxidant activity of edible films

For the DPPH assay, film extract solution was prepared by dissolving 20 mg of ethanolic extract-loaded films in 2 mL of water and mixing 1 mL of the film solution with 0.2 mL of a 1 mM DPPH methanolic solution. The solution was completely mixed and kept in the dark for 30 min at ambient temperature. Afterward, the absorbance of the mixture was measured at 517 nm, relative to the comparable blank solution, using a UV–Vis spectrometer (Annu et al., 2021). The technique was performed three times, and the percentage of DPPH scavenging activity was calculated.


$$\begin{array}{l} \text{DPPH Scavenging activity}\;(\%)=\\ \frac{\text{absorbance of control}-\text{absorbance of sample}}{\text{absorbance of control}}\times 100 \end{array}$$

### 1.17 Antimicrobial activity of edible films

To estimate the antimicrobial effects of each film sample against microorganisms (Akalin et al., 2022), the microorganisms were grown for 24 h, and Mueller Hinton agar was prepared with a uniform distribution of 0.2 mL of a bacterial strain at 10^6^ CFU/mL. The uppermost layer of the agar plate was covered with each film sample, and the antimicrobial plates were kept at 37 °C for 24 h. Finally, the zone of inhibition was measured for the film samples on the plates using a vernier scale

### 1.18 Biodegradability test

Corresponding to the protocol with a few changes, the film samples (5 mg) were combined (5 g) with locally obtained soil without any alteration, disruption, or sterilization to preserve the native microbiota. Every day, 15 mL of water was poured on the film samples for a period of 20 days. The total amount of film samples was determined, and the depletion of soil is denoted as a percentage decrease in weight (Shivangi et al., 2021). The weight reduction was calculated using the following formula:


$$\%\text{weight loss}=\frac{\text{weight loss}-\text{initial weight}\;}{\text{weight loss}}\times 100$$

### 1.19 Evaluation of edible films for preservation of apple slices

The objective of the food preservation test is to estimate the film sample’s capability to retain food products until consumption and adhere to the evaluation technique (Vimala Bharathi et al., 2020). We employed our homemade films to encapsulate the fresh-cut apple slices and eliminate any potential defenses. To determine moisture reduction, we weighed the fresh-cut samples prior to (W_o_) and after (W_f_) and applied the formula.


$$\%\text{Moisture ratio}=\frac{w0-\mathrm{wf}}{w0}\times 100$$

Subsequently, 2 g of samples were combined with 5 mL of distilled water, and then the mixture was centrifuged to produce extract solutions to further determine the total phenolic content (TPC; Annu et al., 2021) and browning index (Jancikova et al., 2021; Subramani and Manian, 2024).

## 2 Results

### 2.1 Percentage yields

As shown in Supplementary Table S1, the yields of *C. halicacabum* leaf extraction with petroleum ether, chloroform, and ethanol were 16.2, 21.5, and 24.2%, respectively. Ethanol obtained the highest yield compared to other solvent extracts, probably because of its polarity; it facilitates the extraction of a range of bioactive compounds such as flavonoids, phenolics, and glycosides. On the other hand, the decreased yield of petroleum ether implies its efficiency in retrieving non-polar materials like lipids and waxes. Conversely, chloroform is considered a rather polar solvent for extracting substances like terpenoids and alkaloids, resulting in an intermediate yield.

### 2.2 Estimation of antioxidants with DPPH assay

**Figure 1:**
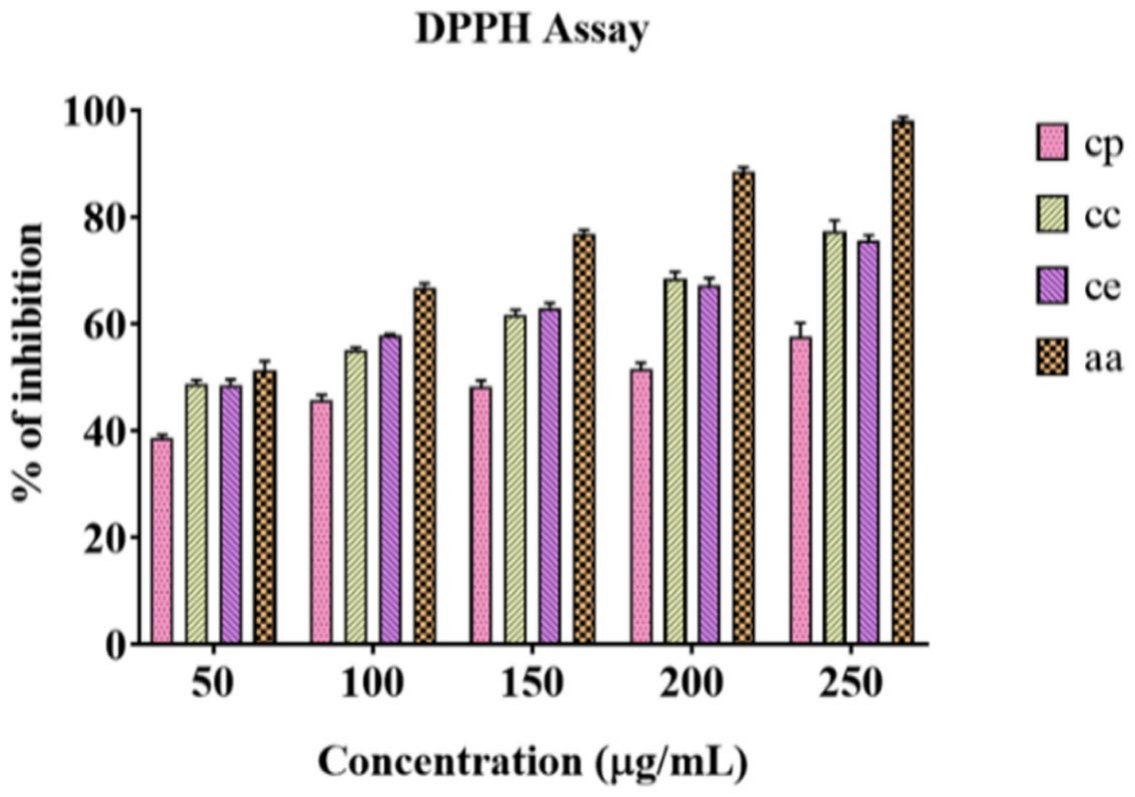
Antioxidant capacity of DPPH radical inhibition activity with various solvent extracts of *C. halicacabum* at different concentrations compared to that of ascorbic acid. CP: *Cardiospermum halicacabum* petroleum ether extract, CC: *Cardiospermum halicacabum* chloroform extract, CE: *Cardiospermum halicacabum* ethanol extract, and AA: Ascorbic acid (standard).

Petroleum ether, chloroform, and ethanol were used to evaluate the DPPH antioxidative potential of *C. halicacabum* extracts, resulting in IC50 values of 205.2 μg/mL, 169.7 μg/mL, and 166.4 μg/mL, respectively. The % inhibition of DPPH free radical generation by the various solvent extracts and standard ascorbic acid is illustrated in Figure 1 and Table 1. According to these findings, the ethanol extract showed the highest antioxidant activity compared to the other extracts, followed by chloroform and petroleum ether extracts. With an IC50 of 117.4 μg/mL, the standard ascorbic acid exhibited the most activity, demonstrating its high efficacy as a free radical scavenger. These findings indicate that the extracts, especially those from ethanol, have a high level of antioxidant potential, whereas chloroform and petroleum ether have a moderate level. This is likely due to their significant amounts of polar phytochemicals such as flavonoids and phenolics. These outcomes support findings from earlier research that highlight the association between solvent polarity and plant extract antioxidant properties (Mansoori et al., 2020). The antioxidant capacity of aqueous and ethanolic extracts of *L. neesiana* stems was demonstrated in our earlier study, with IC_50_ values of 175.32 and 187.91 μg/mL, respectively. It was found that an ethanolic extract of *L. neesiana* leaves and twigs had an IC_50_ value of 29.5 ± 1.04 μg/mL, indicating its antioxidant activity (Brand-Williams et al., 1995; Adhikari-devkota et al., 2019). The two-way ANOVA analysis exhibited a significant interaction between extract and concentration [*F* (12, 40) = 47.83, *p* < 0.0001], revealing an important effect size (η^2^ = 0.064, partial η^2^ = 0.935) and a confidence level of 99.99%.

**Table 1:** DPPH free radical scavenging activity.

DPPH assay				
Concentration μg/mL	Petroleum ether	Chloroform	Ethanol	Ascorbic acid (standard)
50	38.61 ± 0.52	48.72 ± 0.68	48.56 ± 0.86	51.24 ± 1.79
100	45.71 ± 0.81	55.05 ± 0.43	57.84 ± 0.22	66.83 ± 0.76
150	48.25 ± 0.99	61.70 ± 0.74	63.01 ± 0.76	76.94 ± 0.67
200	51.53 ± 0.99	68.42 ± 1.07	67.22 ± 1.10	88.38 ± 0.98
250	57.60 ± 2.10	74.32 ± 1.68	75.54 ± 0.85	97.97 ± 0.80
IC_50_	205.2	169.7	166.4	117.4

Data is shown as mean ± standard deviation from triplicates with their corresponding IC50 values. Statistical significance was evaluated utilizing the two-way ANOVA followed by Tukey’s *post-hoc* test (*p* < 0.05).

### 2.3 Antimicrobial activity of different solvent extracts

**Figure 2:**
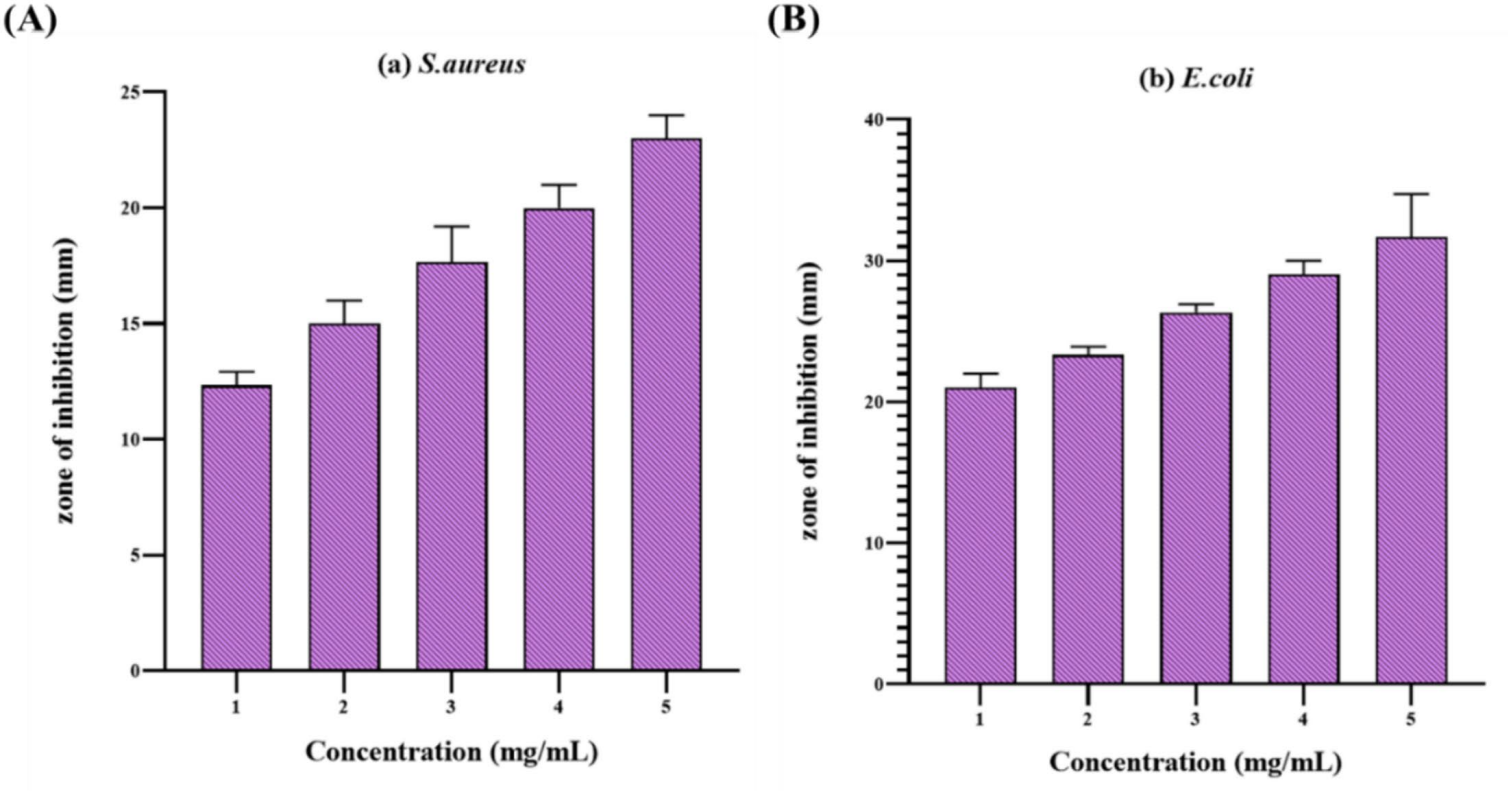
Bacterial growth inhibitory of ethanol extract toward microorganisms **(A)** *S. aureus and* **(B)** *E. coli*.

The well diffusion assay was performed to evaluate the antimicrobial inhibitory effect of *C. halicacabum* extracts toward *S. aureus* and *E. coli* pathogens at different concentrations of 10 to 25 mg/mL. Figure 2 signifies the outcomes of experiments executed on different bacteria to examine the antimicrobial potency of various crude extracts (Table 2). The ethanol extract exhibited strong antibacterial action, with inhibition zones measuring 20 ± 0.81 mm (*S. aureus*) and 29 ± 0.8 mm (*E. coli*) at a dose-dependent level. In comparison, both petroleum ether and chloroform extracts demonstrated absence or less antimicrobial activity against the pathogens. In comparison to the prevalent antibiotic tetracycline, it shows inhibition zones of 20.66 ± 0.47 mm for *S. aureus* and 31.66 ± 2.49 mm for *E. coli*. These results indicate that the ethanol extract contains bioactive substances that are highly efficient toward *S. aureus* and *E. coli*, particularly *E. coli*. These results align with earlier studies that found plant materials to be efficient against both bacterial strains, as polar phytochemicals can break down bacterial membranes. The improved sensitivity of Gram-negative organisms to antimicrobial constituents is possibly due to the lipopolysaccharides that constitute their exterior membrane. The results obtained indicate that broad-spectrum antibacterial substances may be detected in *C. halicacabum*. With the inhibition zones varying between 13.4 to 26.3 mm, it has been shown that the clove ethanol extract has the potential to be highly effective against *S. aureus*, pseudomonads, *L. monocytogenes*, aeromonads, *V. parahaemolyticus*, and *A. faecalis* (Hoque, 2008).

**Table 2:** Antimicrobial activity (zone of inhibition) of different solvent extracts against foodborne pathogens was measured, with values expressed as mean ± standard deviation (*n* = 3).

Concentration mg/mL	Zone of inhibition (mm in dia) diameter					
	*S. aureus*			*E. coli*		
	Petroleum ether	Chloroform	Ethanol	Petroleum ether	Chloroform	Ethanol
10	-	-	12.33 ± 0.47	-	-	21 ± 0.81
15	-	-	15 ± 0.81	-	-	23.33 ± 0.47
20	-	-	17.66 ± 1.24	-	-	26.33 ± 0.47
25	-	-	20 ± 0.81	-	-	29 ± 0.81
Tetracycline (positive)	20.66 ± 0.47	22 ± 0.81	23 ± 0.81	29.3 ± 0.47	29 ± 0.81	31.66 ± 2.49
(DMSO) negative	-	-		-	-	

Differences among groups were analyzed utilizing two-way ANOVA with Tukey’s *post-hoc* test for multiple comparisons (*p* < 0.05).

The ethanolic extract exhibited concentration-dependent antimicrobial efficiency toward *S. aureus* and *E. coli*. The two-way ANOVA revealed substantial main impacts of extract proportion (*F* (1,4) = 206.1, *p* = 0.0001, η^2^ = 0.549, 95% CI [0.31–0.73]) and type of microorganism (*F* (4,16) = 69.98, *p* < 0.0001, η^2^ = 0.416, 95% CI [0.22–0.61]) on the diameter of the inhibition zone. No significant interaction between extract concentration and microorganism type was observed (F (4,16) = 0.055, *p* = 0.994, η^2^ = 0.032, 95% CI [0–0.05]), indicating that the effect of extract concentration was similar across the two pathogens. Post-hoc analysis showed that the 25 mg/mL ethanolic extract produced notably higher inhibition related to minimal proportions toward both pathogens. According to these findings, *C. halicacabum* might have significant potential in the development of a broad range of antimicrobial agents. As predicted, the results of this search validated that the leaf extract of *C. halicacabum* effectively inhibits the growth of microorganisms and exhibits various levels of antimicrobial activity.

### 2.4 Cytotoxicity studies with evaluation of root growth inhibition and chromosomal aberrations

In the area of morphology, the hydroponically developed onion bulb roots performed effectively. Onion roots treated with extract at various concentrations of 25, 50, 75, and 100 μg/mL showed root growth in Supplementary Figure S1. The roots are robust, long, glossy, and dense, growing quickly, all of which are evidence of strong cell division that promotes the root’s growth and development. The plant substances exhibited a concentration-dependent reduction in extent and color, but the *A. cepa* treated with the negative control (tap water) propagated well and produced long white roots (Alabi et al., 2022). The effects of *C. halicacabum* active extract at different concentrations on *A. cepa* root growth inhibition are depicted in Supplementary Figure S1. This study aligns with previous results; *T. bangwensis* and *M. oleifera* extracts and fractions demonstrated cytotoxicity, as shown by a lower percentage of mitotic cells. The number of cells undergoing division and root elongation decreased with increasing concentration (Ihegboro et al., 2020).

**Figure 3:**
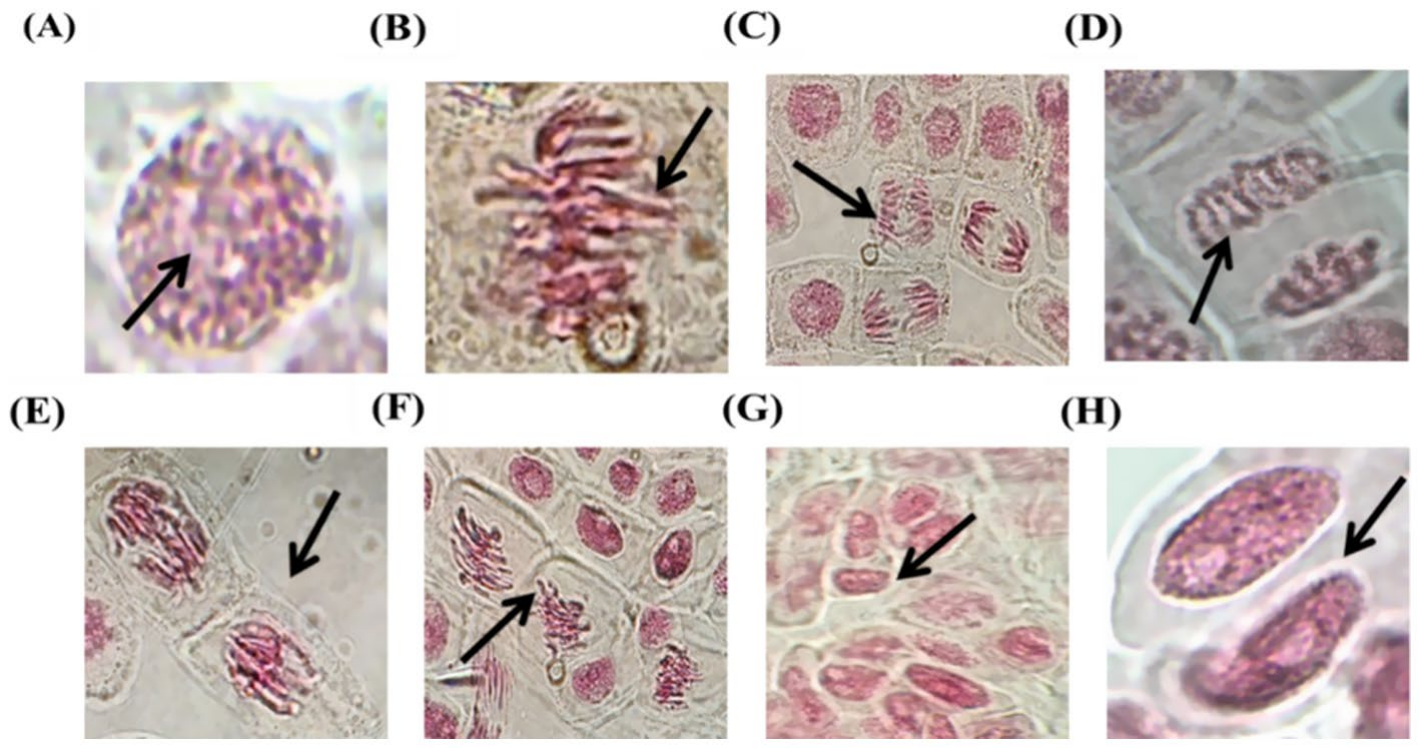
Chromosomal aberrations **(A–D)** Prophase, **(B)** metaphase, **(C)** anaphase, **(D)** telophase **(E)** spindle formation with anaphase **(F)** vagrant chromosome **(G)** binucleated cells **(H)** giant nucleus.

Chromosomal aberrations are changes in the shape of chromosomes caused by mutational conformation and can be caused by UV light, harmful chemicals, medications, herbal medicines, and phytochemical compounds (Başyiğit et al., 2020). A break or exchange of chromosomal materials can cause chromosomal aberrations (CAs), which are modifications in chromosome architecture or structure. The chromosomal abnormalities identified in the present study included delayed/lagging chromosomes, fragments, binucleated cells, and interrupted spindles. Binucleated cells, spindle disruption, sticky chromosomes, delayed (lagging) chromosomes, and fragmented (or shattered) chromosomes constitute some of the abnormalities observed in Figure 3. The images show that various phases of the cell cycle are impacted by a possible toxicant in the cytotoxicity test using *A. cepa* root tips. Images show the different stages of mitosis: (A) prophase: during which chromatin condenses into visible chromosomes, (B) metaphase: during which chromosomes are arranged along the equatorial plate, (C) anaphase: during which chromosomes move to opposite poles, (D) telophase: chromosomes are split into two nuclei, and finally (E-H) observed abnormalities, including chromosome clumping or stickiness and multipolar divisions, imply cytotoxic or genotoxic impacts from the tested compound, along with nuclear aberrations such as micronuclei, which are widely recognized markers of genotoxicity.

According to the results, the analyzed substance interferes with normal cell division, most likely by interacting with the mitotic spindle apparatus or affecting the integrity of DNA, which causes chromosomal abnormalities or mitotic arrest. Because of its association with other bioassays and susceptibility to environmental pollutants, the *A. cepa* test is widely recognized. The reported abnormalities refer to the tested substance’s potential for cytotoxicity and genotoxicity. These effects are consistent with previous studies on genotoxicity and are possibly related to alkaloids and phenolic substances that interfere with mitotic processes. However, the cytotoxicity that occurred highlights the requirement for further study to identify its active ingredients, investigate their mechanisms, and improve safe therapeutic dosages (Alias et al., 2023).

### 2.5 TLC profiling

The ethanol extract was elevated using different solvents, and the best segregation was attained by toluene: ethyl acetate in a 4:1 ratio, as indicated by Rf values illustrated in Supplementary Figure S2 and Supplementary Table S2. In the TLC sheet, phytocomponents evaluated with higher Rf values traveled a greater distance. It comprised a diverse array of phytocomponents, confirming their relative polarity differences and the presence of different phytocompounds, such as phenols and flavonoids.

#### 2.5.1 Screening of fractionation of antimicrobial activity

**Figure 4:**
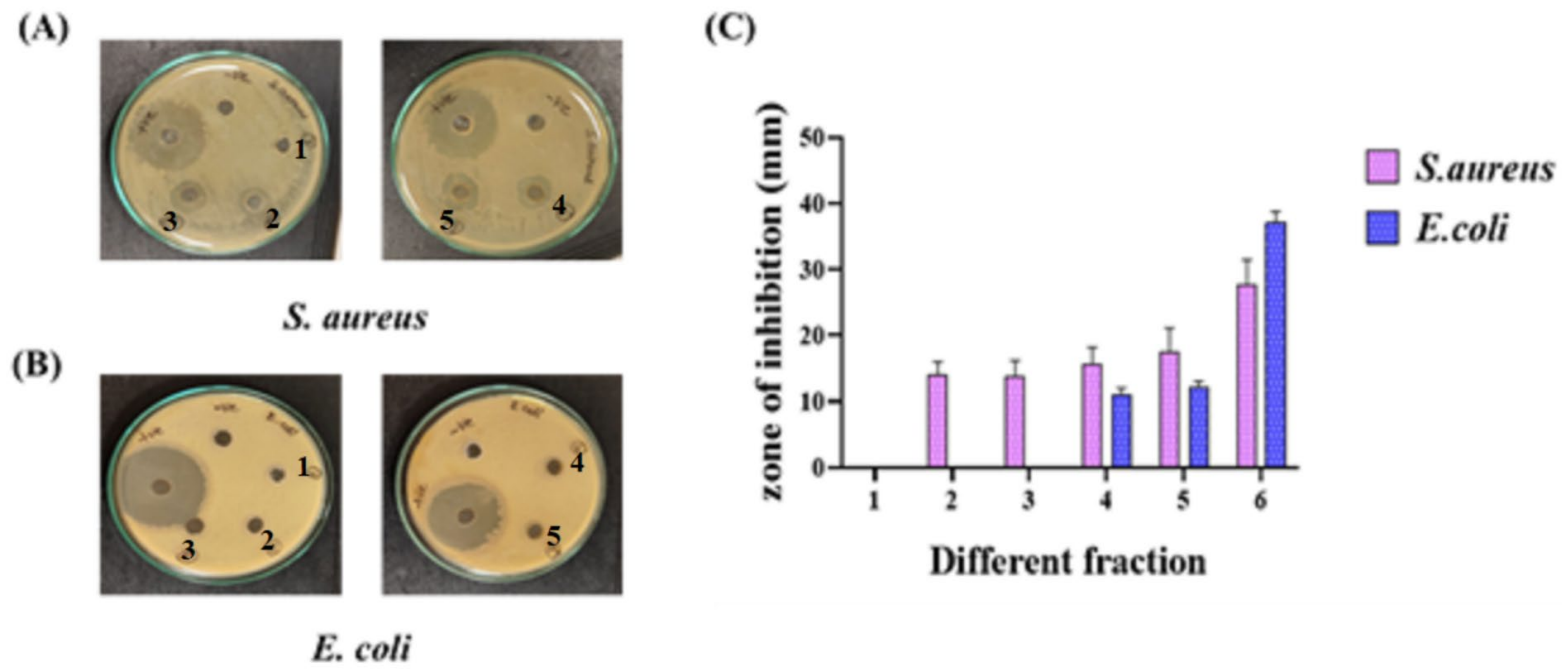
Antibacterial activity of *C. halicabum* ethanol extract of six portions toward foodborne pathogens **(A)** *S. aureus* and **(B)** *E. coli.* **(C)** Quantitativemeasurements of growth inhibition.

Fractions were systematically collected through column chromatography using silica gel and subsequently explored for the identification of different components with TLC. The eluted fractions were correlated based on Rf values, and each peak was measured before evaporation to prevent loss of moisture by utilizing a rotary evaporator. The dried weight of each portion was measured, and the eluted fractions were obtained and subsequently classified based on their TLC results, which indicated equivalent Rf values. The five concentrated portions were labeled from 1 to 5 and assessed for antimicrobial activity. All fractions showed inhibition of growth in *S. aureus* except for fraction 1, whereas *E. coli* showed no inhibition except for fractions 4 and 5. Figure 4 and Supplementary Table S3 demonstrate the analysis of five fractions for antibacterial activity against foodborne microorganisms. Comparing the results, fractions 4 and 5 showed the highest zones of inhibition in *S. aureus* (15 mm and 17 mm) and for *E. coli* (11 mm and 12 mm). We further proceeded with fraction 5 for applications to create an edible film for food preservation and packaging.

### 2.6 GC–MS profiling

**Figure 5:**
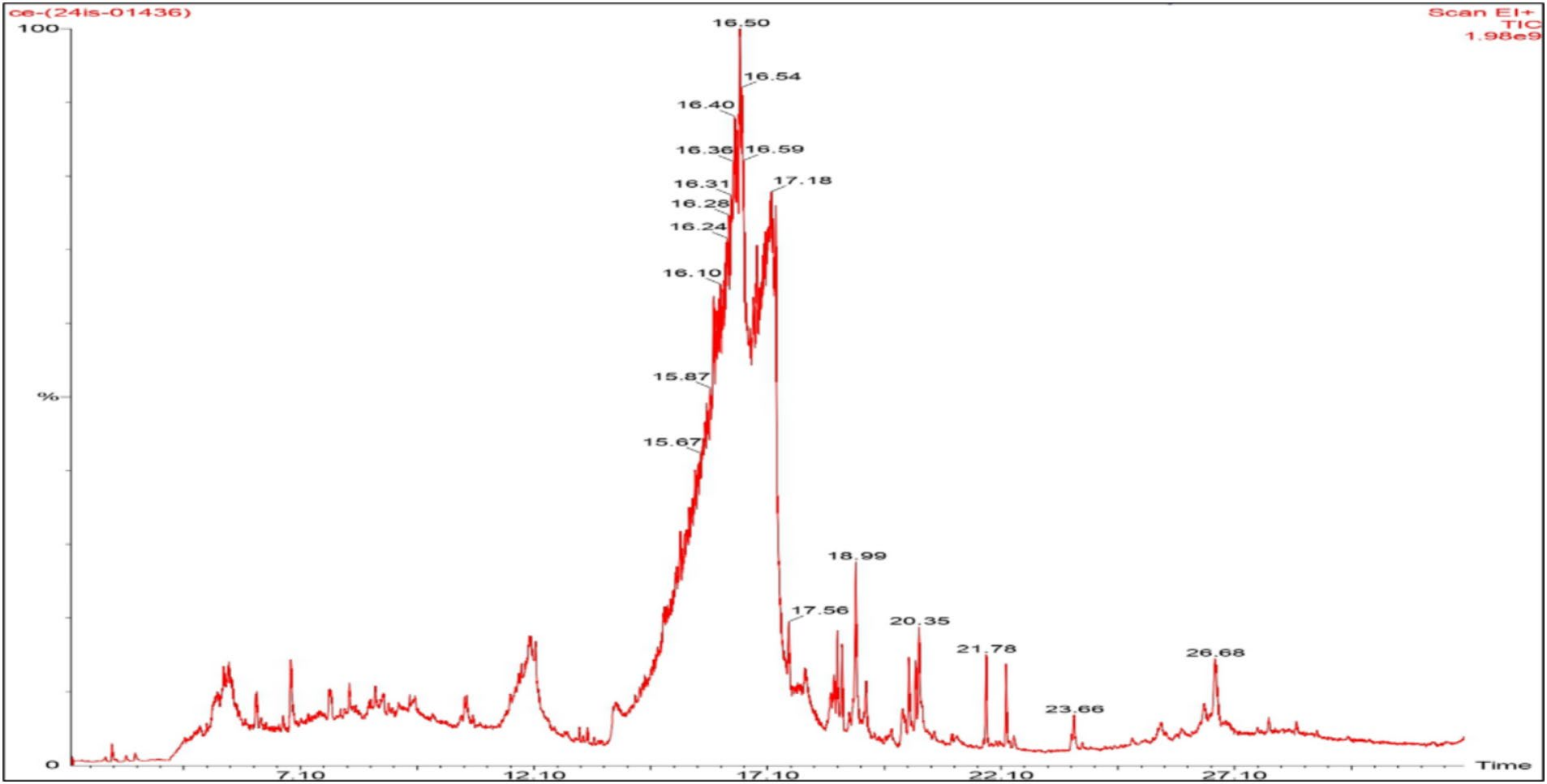
GC–MS chromatogram of *C. halicacabum* fraction to found plant-based compounds.

Previous reports have demonstrated the use of GC–MS analysis for the identification of phytocomponents (Coteli et al., 2024; Venkatesan and Muniyan, 2025). The fractions obtained through TLC were assessed for antibacterial properties, and the extremely bioactive fraction underwent GC–MS exploration for the identification of phytocomponents. Through GC–MS analysis, 27 compounds were identified in the partially purified ethanolic extract of *C. halicacabum*, and these compounds were matched to NIST and Wiley libraries (Supplementary Table S4). Figure 5 represents the GC–MS chromatogram of the partially purified ethanol-based extract of *C. halicacabum* and shows the majority of phytocomponents identified in the extract. From the partially purified ethanolic extract, most of the compounds exhibited various biological properties, and potential applications as food preservatives were identified, including newly reported compounds. These compounds also showed binding affinities in molecular docking analyses. Based on their elevated peak area percentages (representing relative abundance) and previously reported biological activities, the uniqueness of the *C. halicacabum* extract, and these components were considered significant. These techniques assure the involvement of both evidenced and possibly novel biologically active components in the computational estimations. In summary, the GC–MS findings confirm the existence of identified compounds and suggest the existence of new components with potential applications in food preservation and pharmacological studies. Our results align with previous studies reporting the presence of various metabolites in *T. hamosa* seeds, where the availability and bioactivity demonstrated the effectiveness of *in vitro* assays (Rao et al., 2023).

### 2.7 *In silico* docking

The interactions between the phytocomponents and the target proteins were estimated through docking to elucidate the process correlated with the substantial antimicrobial activity of the extract. Based on the GC–MS profiling, a total of 27 phytocomponents were found and retrieved from the PubChem database in SDF formats.^4^ These phytocomponents were docked against the target proteins utilizing AutoDock Vina, and their pharmacokinetic properties were analyzed using Swiss-ADME and Protox tools. It was observed that 27 phytocomponents successfully passed the ADME filters and showed no violations of Lipinski’s rule of five, and their toxicity profiles were evaluated in Table 3. Supplementary Table S5 illustrates the results of the hit compounds estimated through ADME and toxicity studies.

**Table 3:** List of hit compounds that passed the Lipinski rule of five.

Hits / Reference compound	Molecular weight	PubChem ID	Lipinski	GI absorption	BBB permeant	#H-bond acceptors	#H-bond donors	PAINS #alerts	LogP	TPSA (Å^2^)
Hit 1	279.46	534,426	0 violation	High	Yes	1	1	0	4.42	43.09
Hit 2	298.25	135,599,155	0 violation	low	No	8	5	0	−1.91	153.72
Hit 3	296.24	638,122	0 violation	low	Yes	8	4	0	−1.62	150.56
Reference 1	366.5	9,543,473	1 violation	Low	No	4	6	0	1.02	227.10
Reference 2	408.25	54,759,160	0 violation	High	No	4	2	0	1.92	106.84

**Figure 6:**
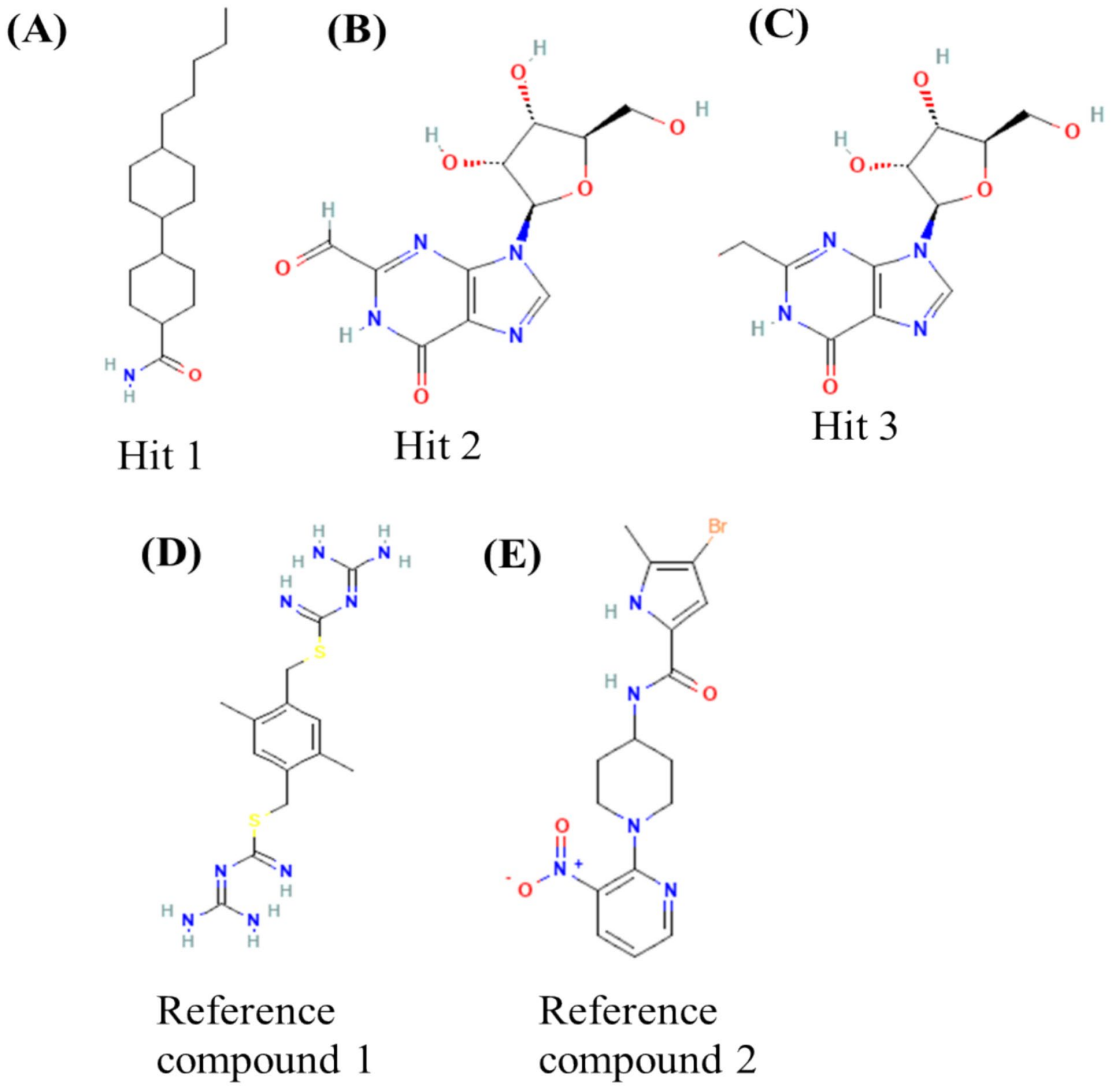
Molecular structures of identified references and hits **(A)** Hit 1, **(B)** Hit 2, **(C)** Hit 3, **(D)** Reference compound 1, and **(E)** Reference compound 2.

**Figure 7:**
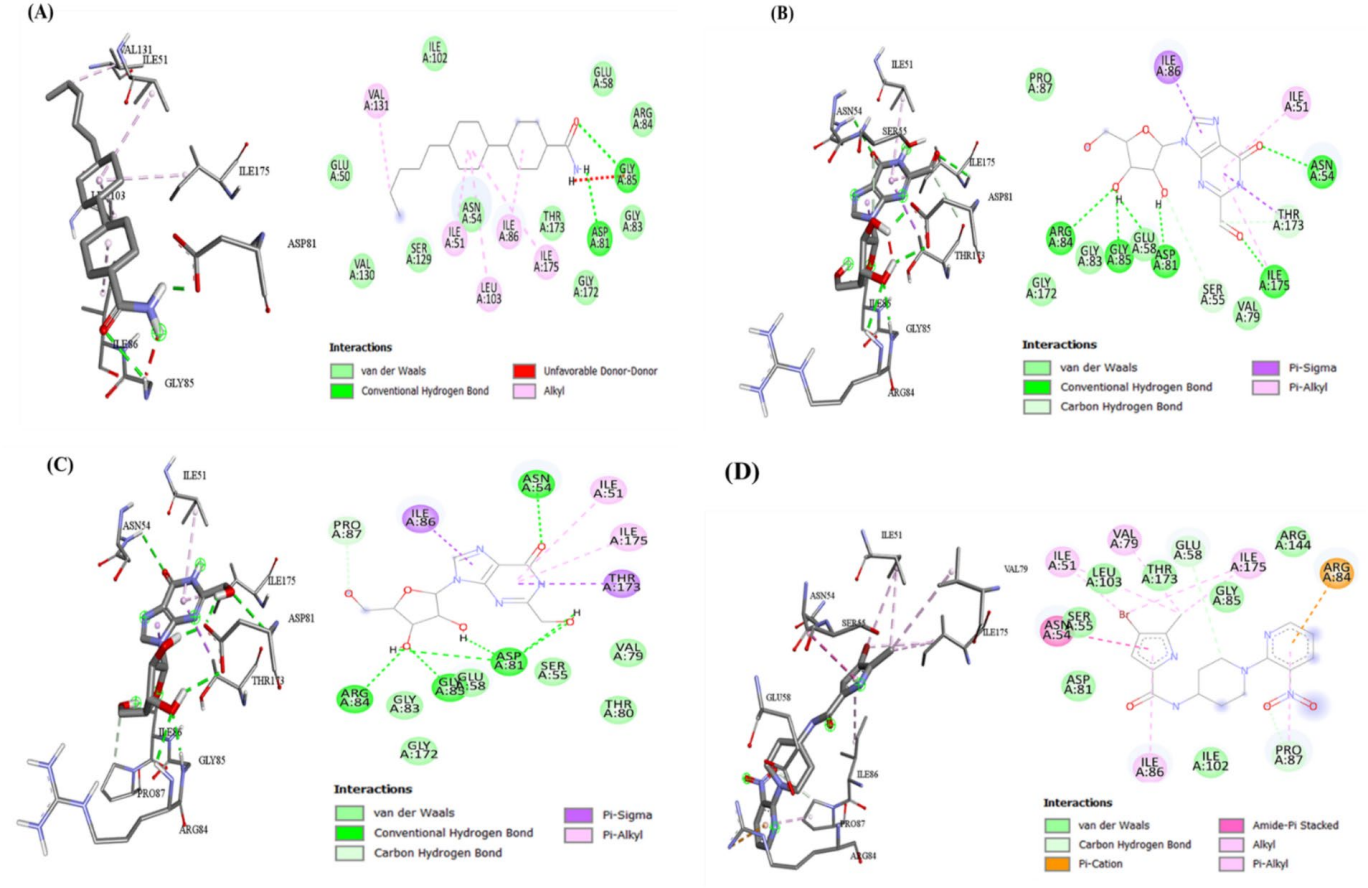
Top-docked structures of GC–MS components with microbial protein of GyrB with 3D and 2D molecular interaction of **(A)** Hit 1, **(B)** Hit 2, **(C)** Hit 3, and **(D)** Reference compound 1.

**Figure 8:**
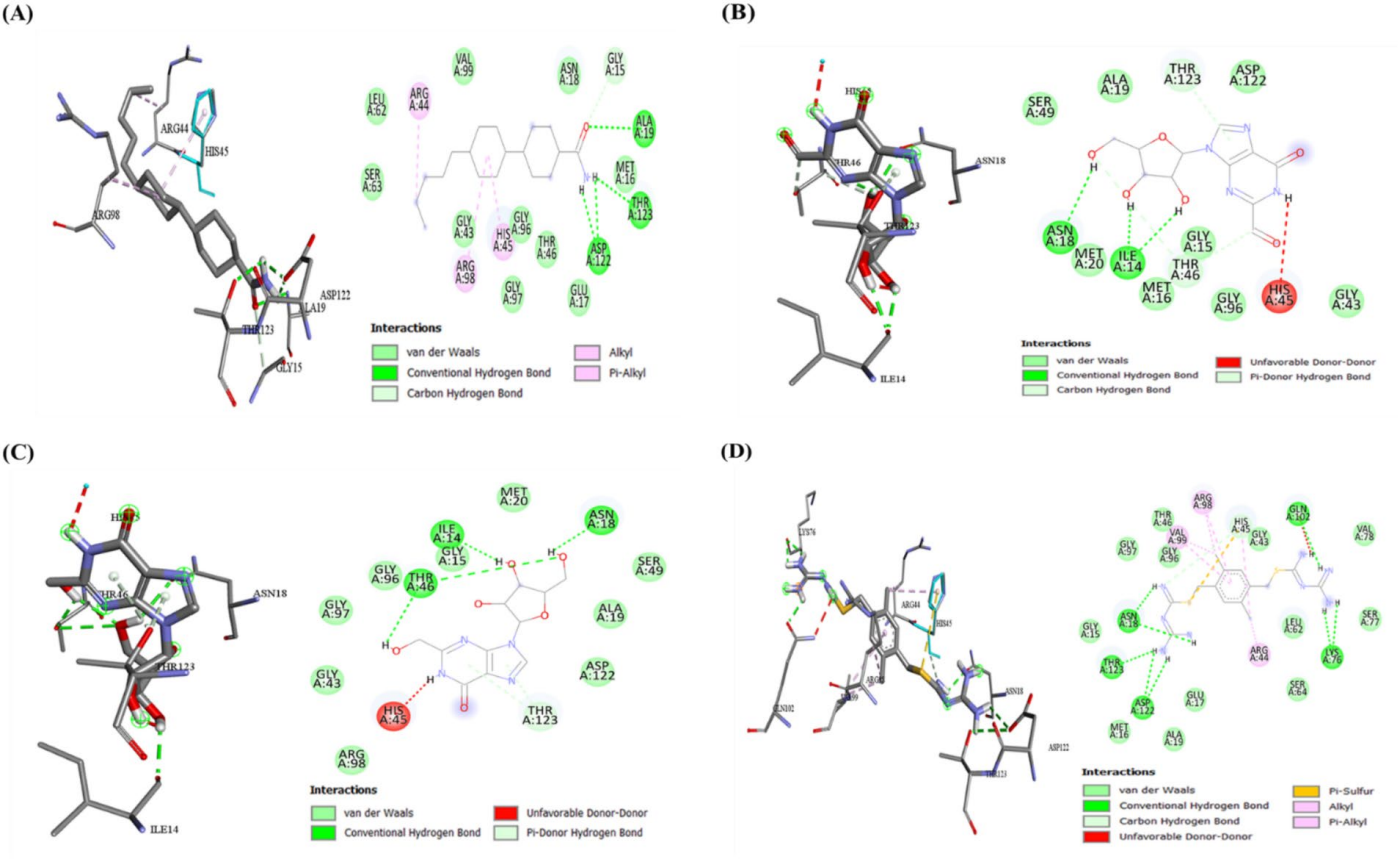
Top-docked structures of GC–MS components with microbial protein DFHR with 3D and 2D molecular interaction of **(A)** Hit 1, **(B)** Hit 2, **(C)** Hit 3, and **(D)** Reference compound 2.

The preliminary screening was done using the Autodock Vina tool; subsequently, the compounds that exhibited superior behavior were subjected to individual protein–ligand docking in the Autodock tool with 100 iterations. The detailed docking outcomes, including binding energies, interactions with amino acid residues, total number of hydrogen bonds, and exhaustive docking scores, are illustrated in Table 4. The docking results revealed the binding energies for GyrB and DFHR: −9.57 and −16.33 kcal/mol for Hit 1, −10.95 and −15.00 kcal/mol for Hit 2, −11.18 and −14.80 kcal/mol for Hit 3, and −13.22 and −6.27 kcal/mol for Reference compounds 1 and 2, respectively. Through the post-docking evaluation, the top three components were identified based on the docking score and key amino acid interactions and contrasted with their respective reference components against the target proteins. The molecular structures of the identified hit components and reference ligands are depicted in Figure 6. Figures 7, 8 illustrate both the 2D and 3D molecular docking interactions of the hit components and reference ligands with the target proteins. Based on the in-silico analysis, it can be inferred that the compounds present in the partially purified extract, particularly these three selected hits, possibly contribute to the antimicrobial activity of the extract. These hit compounds were further subjected to simulation studies along with protein targets; redocking of the reference ligands duplicated the binding poses within the RMSD tolerance of 2.0 Å, thus confirming the dependability and stability of the docking protocol. Overall, more negative docking scores indicate greater binding affinities and more persistent ligand–protein interactions (Bhagyanath et al., 2026).

**Table 4:** Screened phytocomponents toward target proteins of DNA gyrase B (GyrB) from *S. aureus* and dihydrofolate reductase (DHFR) from *E. coli* by docking study.

Pubchem id / Hits	Name and structure of compounds	GyrB					DFHR				
		Vina score	Exhaustive score	Cluster size	Conventional Hbonds	vanderwaals	Vina score	Exhaustive score	Cluster size	Conventional Hbonds	vanderwaals
534,426 / Hit1	4’-Pentylbicyclohexyl-4-carboxamide 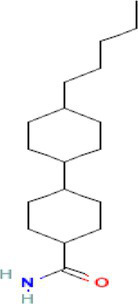	−6.7	−9.57	71	asp-81 gly-85	ile-102 glu-50 val-130 ser-129 asn-54 thr-173 gly-172 gly-83 glu-58 arg-84	−7	−16.33	77	ala-19 thr-123 asp-122	asn-18 val-99 leu-62 ser-63 gly-43 gly-96 gly-97 thr-46 glu-17 met 16
135,599,155 / Hit2	2-Hydroxymethyl-9-[beta-d-ribofuranosyl] hypoxanthine 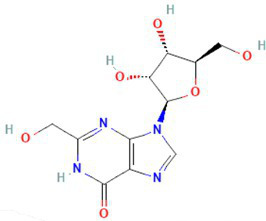	−6.3	−11.18	85	arg-84 gly-85 asp-81 asn-54	gly-83 gly-172 glu-58 ser-55 val-79 thr-80	−6.7	−14.8	23	asn-18 ile-14 thr-46	asp-122 ala-19 ser-49 met 20 gly-15 gly-96 gly-97 gly-43 arg-98
135,599,166 / Hit3	2-Formyl-9-[beta-d-ribofuranosyl] hypoxanthine 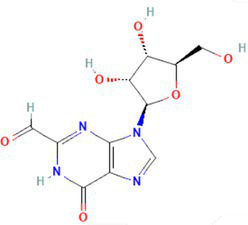	−6.3	−10.95	97	arg-84 gly-85 asp-81 ile-175 asn-54	gly-83 glu-58 pro-87 gly-172	−7	−15	7	asn-18 ile-14	asp-122 ala-19 ser-49 met 20 met 16 gly-15 gly-96 gly-43
Reference 1 and Reference 2	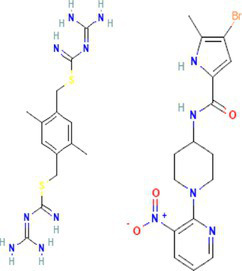	-	−13.22	4	Thr46, Leu62, Ile94, Gly96, Tyr100	Ala6, Ile14, Gly15, Asn18, Met20, Gly43, His 45, Ser49, Ser63, Gly95, Gly97, Val99, Gln102, Thr123; Alkyl bond: Arg98, Val99	-	−6.27	46	Tyr141, Lys163, Arg198	Trp49, His150, Phe160, Val165, Leu202, Leu205; Alkyl bond: His46

### 2.8 MDS analysis

Subsequently, probing with the post-docking analysis of the selected hit components, molecular dynamics simulations (MDS) were conducted to estimate the constancy, binding mode, and dynamic behavior of the receptor–ligand complexes to provide deeper insights into the interactions (Kato et al., 2021). The reference complexes (1 and 2), along with the top three docked ligands, were executed for 200 ns, and the results were systematically evaluated. Moreover, the progression of the MD simulation was analyzed using RMSD, RMSF, Rg, SASA, and hydrogen bond analysis to evaluate the spatial features of the protein–ligand complex and to assess both flexibility and stability.

#### 2.8.1 Root-mean-square deviation assessment of structural stability

**Figure 9:**
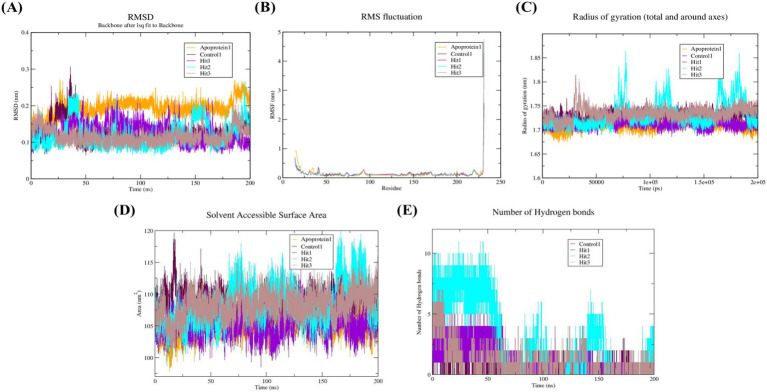
Analysis of molecular dynamics trajectories of GyrB protein and receptor–ligand complexes during 200 ns simulation **(A)** RMSD plots of Apoprotein 1, Reference 1, Hit 1, Hit 2, and Hit 3; **(B)** RMSF plots of Apoprotein 1, Reference compound 1, Hit 1, Hit 2, and Hit 3; **(C)** R_g_ plots of Apoprotein 1, Reference compound 1, Hit 1, Hit 2, and Hit 3; **(D)** SASA plots of Apoprotein 1, Reference compound 1, Hit 1, Hit 2, and Hit 3; and **(E)** Hydrogen bonds plots of Reference compound 1, Hit 1, Hit 2, and Hit 3.

**Figure 10:**
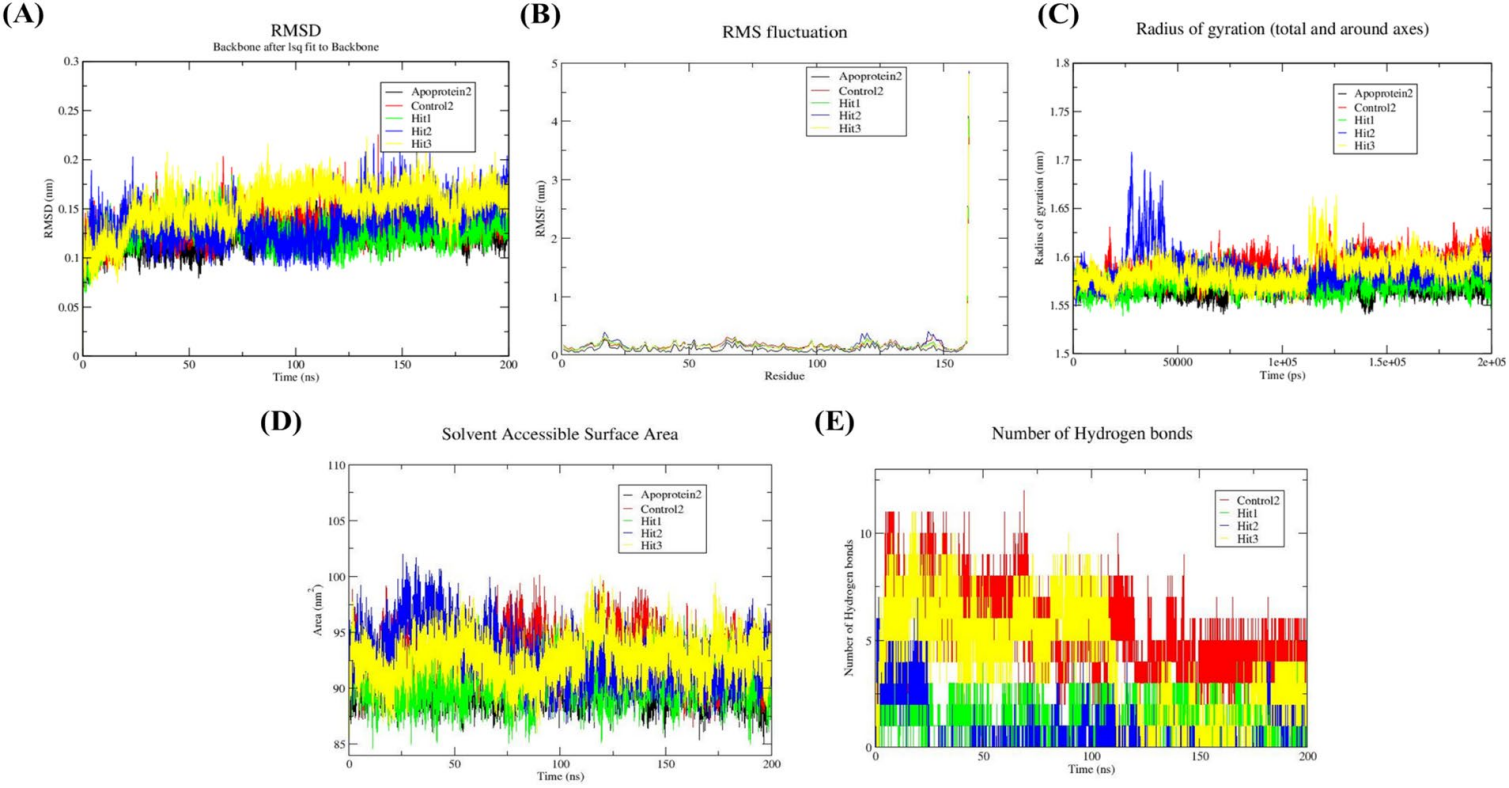
Analysis of molecular dynamics trajectories analysis of DFHR protein and receptor–ligand complexes during 200 ns simulation **(A)** RMSD plots of apoprotein 2, reference compound 2, Hit 1, Hit 2, and Hit 3 **(B)** RMSF plots of apoprotein 2, reference compound 2, Hit 1, Hit 2, and Hit 3 **(C)** Rg plots of apoprotein 2, reference 2, Hit 1, Hit 2, and Hit 3 **(D)** SASA plots of apoprotein 2, reference compound 2, Hit 1, Hit 2, and Hit 3 **(E)** Hydrogen bonds plots of reference compound 2, Hit 1, Hit 2, and Hit 3.

MD simulation was performed to investigate the adaptability and structural stability of the apoprotein and protein–ligand complexes, as well as to acquire the average values (Naithani and Guleria, 2024). These results are essential for understanding the structure of proteins, their functions, and dynamics, and they assist in elucidating the biological activities and development of therapeutic strategies. Figures 9A, 10A represent the protein complexes of both GyrB and DFHR, respectively. Furthermore, Table 5 depicts the mean values of all docked complexes along with their corresponding proteins. For the GyrB protein, Hit 3 exhibited greater structural stability than Hits 1 and 2 when compared with the reference compound. In the case of the DFHR protein, Hit 1 demonstrated higher stability than Hits 2 and 3 relative to the relevant reference compound.

**Table 5:** Mean values of hit components toward the target proteins.

Protein dynamics parameter	GyrB					DFHR				
	Hit 1	Hit 2	Hit 3	Reference 1	Apoprotein 1	Hit 1	Hit 2	Hit 3	Reference 2	Apoprotein2
RMSD	0.117 ± 0.04	0.118 ± 0.03	0.115 ± 0.02	0.126 ± 0.02	0.192 ± 0.02	0.124 ± 0.013	0.135 ± 0.019	0.152 ± 0.021	0.133 ± 0.014	0.120 ± 0.012
RMSF	0.082 ± 0.056	0.081 ± 0.06	0.080 ± 0.05	0.077 ± 0.061	0.095 ± 0.09	0.074 ± 0.030	0.088 ± 0.053	0.080 ± 0.036	0.080 ± 0.041	0.068 ± 0.028
Rg	1.718 ± 0.008	1.716 ± 0.009	1.715 ± 0.009	1.720 ± 0.00	1.711 ± 0.01	1.568 ± 0.008	1.572 ± 0.008	1.570 ± 0.008	1.570 ± 0.008	1.567 ± 0.007
SASA	106.693 ± 1.85	107.012 ± 1.98	105.943 ± 1.87	109.022 ± 1.93	105.734 ± 1.92	90.99 ± 1.62	90.68 ± 1.80	91.12 ± 1.64	90.75 ± 1.60	90.446 ± 1.40
Hydrogen bonds	0 to 5	0 to 11	0 to 7	0 to 3	-	0 to 3	0 to 6	0 to 11	0 to 11	-

#### 2.8.2 Assessment of structural flexibility using RMSF

This analysis elucidates the quantification of the movement of key amino acid residues, and related parameters reflect the structural integrity and flexibility of the protein. A low RMSF value (< 0.1 nm) indicates stiffness and consistent interactions with the respective protein and stability. In contrast, a higher RMSF value (> 1.0 nm) indicates disrupted regions, increased flexibility, and possible accumulation (Bonet et al., 2021). The mean RMSF values of the docked complexes are estimated in Figures 9B, 10B and summarized in Table 5. For the GyrB protein, Hit 1, 2, and 3 exhibited comparable fluctuations (below 0.6 Å), whereas for the DFHR protein, Hit 1, 2, and 3 showed minimal atomic fluctuations (below 0.3 Å), thereby verifying the stable interactions within the binding pocket.

#### 2.8.3 Radius of gyration evaluates compactness

Rg is a crucial parameter for estimating the compactness and overall size of biomacromolecules, including proteins. Lower Rg values indicate greater compactness and a more spherical structure, while higher values denote an enlarged or partially disordered conformation (Bhagyanath et al., 2026). The mean Rg values, calculated based on the intrinsic dynamics of the receptor–ligand complexes, are represented in Figures 9C, 10C, with corresponding values depicted in Table 5. For the GyrB protein, Rg values fluctuated slightly between 1.70 and 1.75 nm, indicating that the ligand-bound protein retained a consistent level of compactness and structural stability throughout the simulation trajectory. Similarly, for the DFHR protein, Rg fluctuations were observed between 1.55 and 1.6 nm, further confirming the stability of the protein–ligand complexes.

#### 2.8.4 SASA analysis

This is a crucial dynamic parameter used to examine the surface area of a molecule exposed to a solvent, which is feasibly related to protein stability. Lower SASA values are linked with enhanced compactness and increased structural stability of the protein (Lee et al., 2014). The SASA plots are presented in Figures 9D, 10D, and the corresponding values are summarized in Table 5. Solvent exposure plays a major role in the folding process and receptor–ligand interactions. In both proteins, the hit compounds exhibited lower SASA values compared to those of the reference compounds, indicating greater stability of the hit complexes along with their respective reference compounds throughout the simulation trajectory.

#### 2.8.5 Evaluation of hydrogen bonds

Estimation of hydrogen bonds supplies crucial insights into molecular binding interactions, binding efficacy, and structural stability (Baba and Malik, 2015). During the 200 ns simulation trajectory, protein–ligand hydrogen bond interactions were observed and represented in Figures 9E, 10E; the corresponding values are depicted in Table 5. For the GyrB protein, Hits 1, 2, and 3 exhibited a higher number of hydrogen bonds compared to the reference compound. In contrast, for the DFHR protein, Hits 1, 2, and 3 showed a comparable number of hydrogen bonds to the respective reference compound.

Overall, *in silico* studies show that Hit 3 for *S. aureus* and Hit 1 for *E. coli* proteins have better binding affinity and may exhibit favorable biological activity as efficient inhibitors.

### 2.9 Visual appearance of various edible films

**Figure 11:**
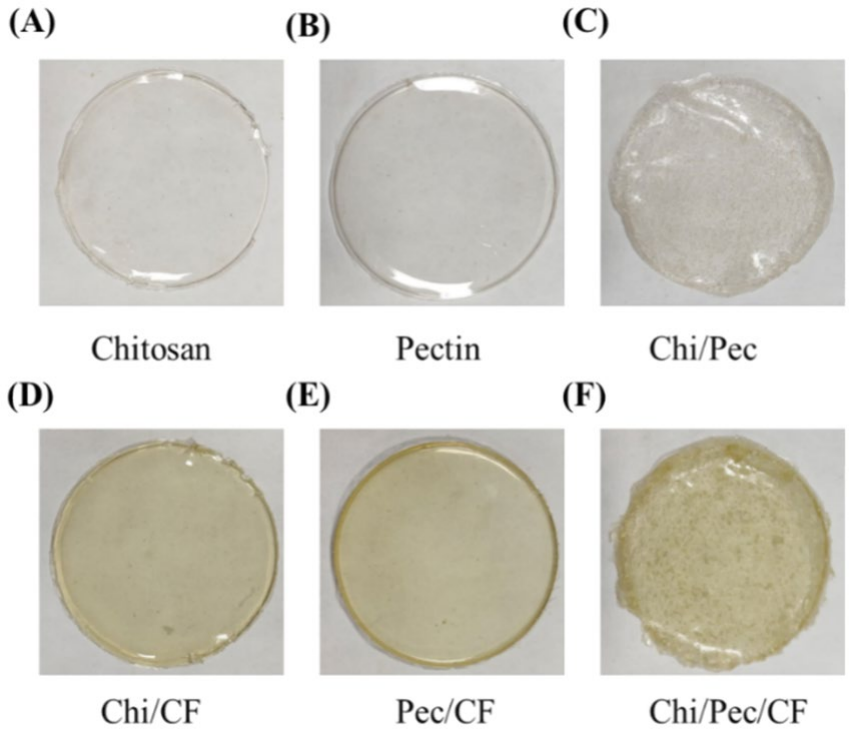
Physical appearance of film samples **(A)** chitosan, **(B)** pectin, **(C)** Chi/Pec, **(D)** Chi/CF, **(E)** Pec/CF, and **(F)** Chi/Pec/CF.

**Figure 12:**
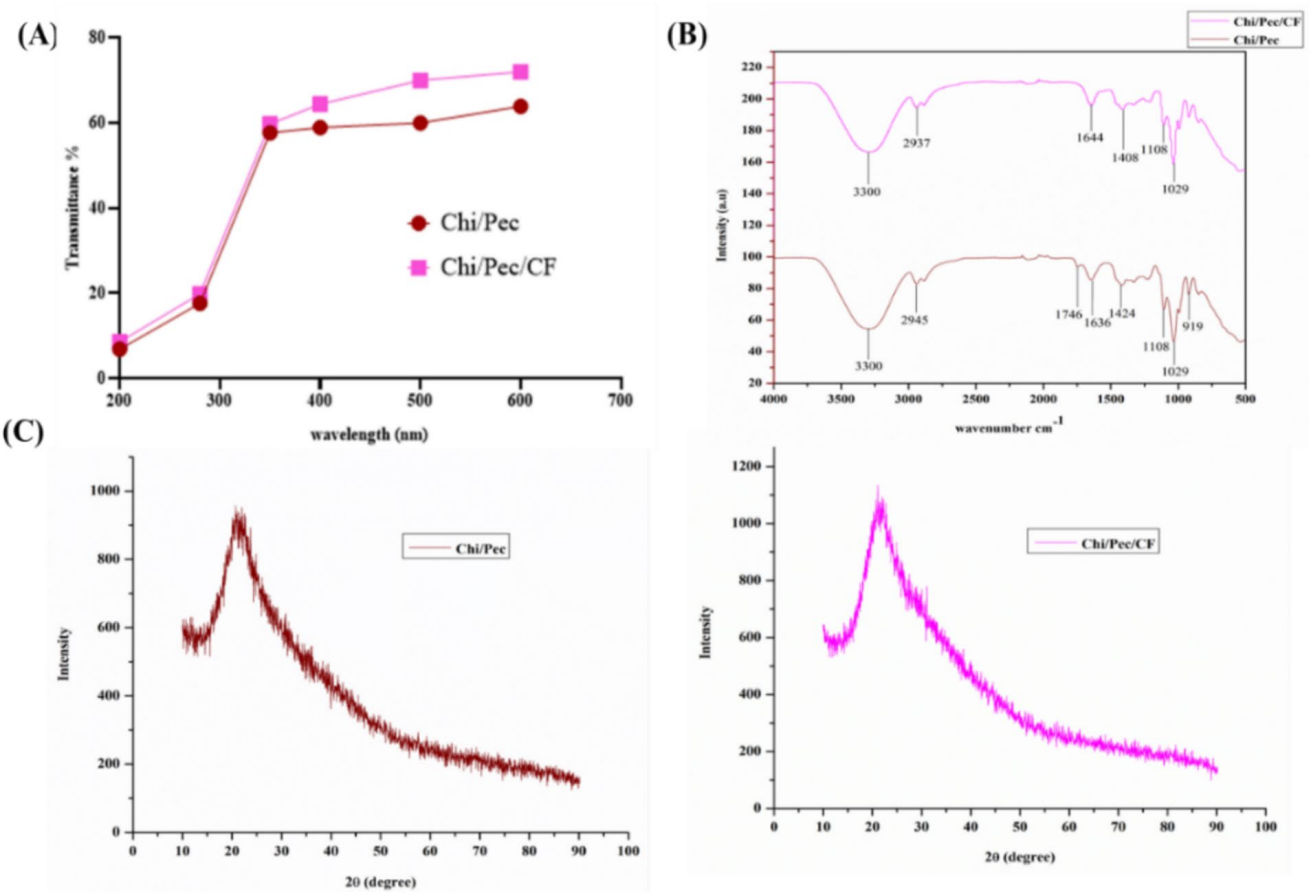
**(A)** UV–Vis spectra of pure chitosan and pectin without plant extract and chitosan–pectin blended films, **(B)** FTIR spectra of Chi/Pec and Chi/Pec/CF films, and **(C)** XRD patterns of films.

The desirable appearance of food wrappings is important for consumer acceptance. Both control films and blended films exemplified transparency, smoothness, and absence of flaws, including consistency, no pores, wrinkles, air bubbles, or cracks, as shown in Figure 11. For optical transmittance, each film was assessed for its visual characteristics by implementing ultraviolet light transmittance of both control and blended films and analyzed in the wavenumber variation of 200–700 nm, confirming the ability of the entire films to block ultraviolet light, as shown in Figure 12A.

### 2.10 Thickness and density

Thickness is one of the main physical properties of films, which allows them to endure extrinsic factors. Generally, an edible film thickness of less than 0.3 mm is often required to ensure efficient food protection (Ribeiro et al., 2021). As shown in Table 6, the inclusion of plant extracts reduced the thickness of films compared to the control films. These results align with previous studies where the chitosan film with ethanolic extract showed a decreased thickness (0.30 mm) compared to the control film (0.78 mm; Karkar et al., 2023). Furthermore, the Chi/Pec/CF film shows higher density due to its more compact structure, indicating greater interaction between the fraction and polymer than the control film in Table 6. Previous studies also reported the same phenomenon, where chitosan films with extracts had a higher density, indicating stronger binding and a highly compact structure.

**Table 6:** Opacity, thickness, and density of both control film and blended films that are blended with bioactive components.

Sample	Opacity (mm^−1^)	Thickness (mm)	Density (g/cm^−3^)	Tensile strength	Elongation break
Chi/Pec control	0.47 ± 0.02	0.82 ± 0.01	2.25 ± 0.05	12.37 ± 0.31	35.92 ± 0.34
Chi/Pec/CF	1.44 ± 0.09	0.28 ± 0.02	6.60 ± 0.12	19.87 ± 0.41	24.51 ± 0.63

### 2.11 Moisture content, solubility and swelling analysis

Another main parameter is moisture content, which influences the functional properties of each film sample. As shown in Table 7, the Chi/Pec/CF matrix had a lower moisture content than the control films, indicating an effective moisture barrier to preserve food products. Similar reports observed that the plant extract-loaded films had decreased moisture content, enhancing the moisture and gas barrier, which is the most important factor in coated food products against surrounding conditions (Martins et al., 2012). Water solubility is a crucial characteristic, specifically in films used in the food industry, providing an understanding of the water resistance of these films (Erken et al., 2022). Table 7 shows the estimation of water solubility in control films and their matrices. Film solubility mainly depends on its features and the concentration of components, as well as the hydrophilic and hydrophobic index. The solubility of the film is improved with the addition of hydrophilic compounds, while it is reduced in the presence of cross-linking agents and hydrophobic substances. The swelling rate plays an essential role in innovative packaging technology due to the components involved in the package, which come from the exterior and modify the package’s design; therefore, an excessive swelling rate negatively impacts the package’s shelf life. As presented in Table 7, composite films showed a reduced swelling index upon extract incorporation, indicating lower water absorption capacity. This reduction is linked to the decreased availability of hydrophilic groups in the modified film matrix (Nisar et al., 2022; Karkar et al., 2023).

**Table 7:** Moisture content, solubility, swelling degree, and water vapor permeability of control and blended films.

Sample	Moisture (%)	Solubility (%)	Swelling degree (%)	Water vapor permeation (x 10 ^−11^ gs^−1^m ^−1^ Pa^−1^)
Chi/Pec control	22.69 ± 0.97	23.12 ± 1.00	21.74 ± 0.90	5.89 ± 0.53
Chi/Pec/CF	20.63 ± 1.07	16.84 ± 0.82	17.60 ± 1.09	4.86 ± 0.09

### 2.12 Water vapor permeation test

It indicates the efficiency of safeguarding films against moisture vapor in packaging materials that have lesser permeability, which is preferable for desired packaging. The permeability of a film depends on the structure of its chemicals and shape, the properties of the permeant, and the surrounding temperature (Erken et al., 2022). As shown in Table 7, extract incorporation significantly reduced WVP in composite films. By minimizing the dispersion of the polymeric chain, it results in decreased development of water vapors through the interaction with the polymer chains and leads to a reduction in water vapor permeability. Similar research indicated a reduction in water vapor permeability in their investigation of chitosan containing black plum extract and TiO_2_ (Zhang et al., 2021).

### 2.13 Mechanical properties of edible films

The mechanical properties of all films, including tensile strength and elongation at break, are illustrated in Table 6. The film samples enriched with the bioactive fraction substantially improved tensile strength, while elongation at break decreased, and there was significant variation in tensile strength compared to the control film. The results indicate that there are very strong interactions between the hydrogen bonds and functional groups of polymer substances, including carboxyl and hydroxyl groups, that provide a formation of a compact structure and increase the tensile strength in the film matrix, consistent with earlier reports (Shivangi et al., 2021). The elongation at break indicates that the fraction incorporated into the films has low resistance and high elasticity compared to the control film. The interactivity of bioactive substances with the plasticizer demonstrated effective effects on the stress related to the polymer (Mohammadian et al., 2021).

### 2.14 Structural analysis of edible films

#### 2.14.1 FTIR analysis

To elucidate the interactions between molecules and alterations in structure in the thin films at the level of molecular structure (Shivangi et al., 2021), the FT-IR technique was used to estimate the interactions of Chi/Pec and Chi/Pec/CF films, as displayed in Figure 12B. The first peaks developed with strong broadband in the wavelength ranges of 3,298–3,333 cm^−1^, which indicates the existence of a hydroxyl group characterized by O-H stretching vibrations that are comparable to those reported earlier (Shivangi et al., 2021). The second peaks were found in the ranges of 2,925–2,876 cm^−1^, which are attributed to C-H stretching of alkane groups (Medeiros Silva et al., 2020; Karkar et al., 2023). The third peak ranges of 1,643–1,648 cm^−1^ represent the amide I band (Annu et al., 2021; Shivangi et al., 2021), O-H stretching, and phenolic groups at 1329 cm^−1^ (Subramani and Manian, 2024), -CO stretching band at 1034 (Rodrigues et al., 2020), and C-I stretching at 849 cm^−1^ (Subramani and Manian, 2024). Based on these results, we conclude that the control film and film matrix produced similar peaks with different amplitudes in bands, and hence the infusion of plant extract does not alter the structure of the films. Furthermore, the prepared edible film was characterized by FTIR spectra; the interactivity of plant extract films was greater and more substantial compared to the control films.

#### 2.14.2 X-ray diffraction

XRD is an influential method to evaluate the structural characteristics of crystalline materials or the molecular configuration of a substance, which is crucial for comprehending its properties and feasible processing concerns. Figure 12C shows the XRD patterns of Chi/Pec and Chi/Pec/CF films. All the films exhibited amorphous properties; the crystalline nature of a polymer is frequently estimated by the identification of prominent peaks, whereas the amorphous characteristics are indicated by the exclusion of such sharp peaks. It indicates that the Chi/Pec film exhibits 2θ ≈ 20.77 and the Chi/Pec/CF film at 2θ ≈ 20.70, with broad peaks and their amorphous nature. The incorporation of CF fraction in the films did not significantly modify the peak positions in the XRD pattern. Nonetheless, the peak amplitude in the XRD results of the blended film exhibited a small reduction relative to the control film. To ascertain that the CF fraction was accurately diffused into the biopolymer matrix and to associate specific functional groups of chitosan, a review of the literature indicates that the 2θ values for pectin correspond to 13° and 22°, which reveal stronger and broader peaks (Akalin et al., 2022). Comparable findings indicate that the control measured 20.66, while the blended films reported at 20.32. The PB leaf extract was precisely incorporated into a biopolymer matrix, enhancing interactions between the phenolic constituents of the leaf extract of PB and the functional groups of chitosan (Nguyen et al., 2022). Hence, it is concluded that the fraction included in the Chi/Pec film did not affect the amorphous structure of the films.

#### 2.14.3 Nuclear magnetic resonance

**Figure 13:**
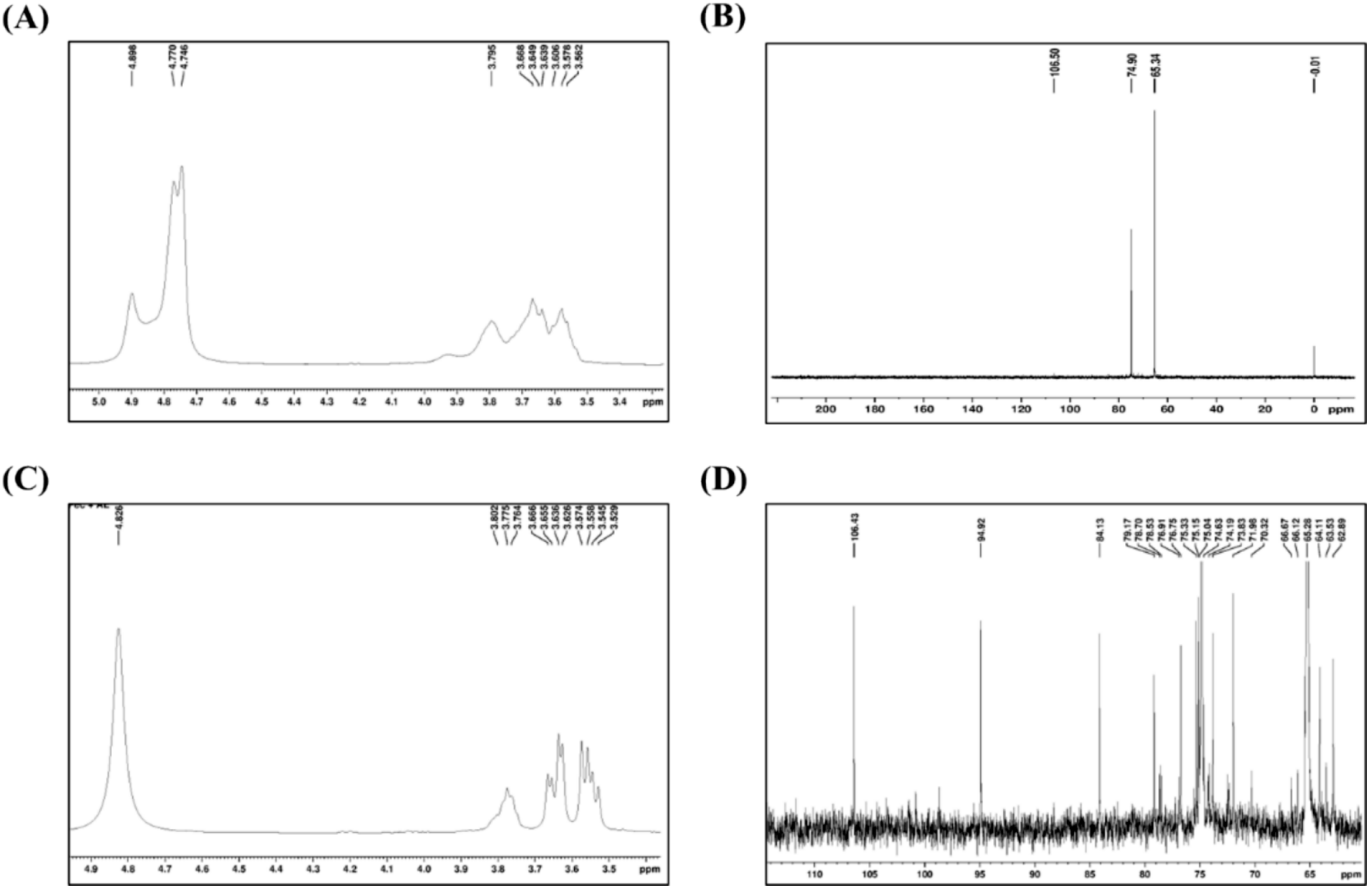
Nuclear magnetic resonance of spectra **(A)** ^1^H NMR spectrum of Chi/Pec film, **(B)** ^13^C NMR spectrum of Chi/Pec film, **(C)** ^1^H NMR spectrum of Chi/Pec/CF film, and **(D)** ^13^C NMR spectrum of Chi/Pec/CF film.

H^1^ NMR and ^13^C analysis further validate the structural conformity of edible films, as illustrated in Figure 13. This typically contributes to the typical chemical alterations of carbohydrate molecules (about 3.0–5.3 ppm), which remain unmodified due to comparatively fewer interactions (Sun et al., 2017). The signals were detected in the 3.5 to 3.8 ppm range, signifying the presence of carbohydrate chains in the polysaccharide complex with active fractions. The subsequent signal at 3.57 ppm is connected to the protons at the 2-position of the glycosamine chain. The signals found at 3.78 and 3.81 ppm are related to the H-6 of glucose units B and C with *α* (1–4) links. The signals that range between 3.58 and 4.83 ppm correspond to a signal in the galacturonic acid units. Compared with previous results, the findings at *δ* 4.3 to 4.8 ppm have been attributed to the *β*-anomeric proton in the β-D-galactose structural unit, indicating the existence of both α-glycosidic and β-glycosidic interactions in the *P. yezoensis* polymer. This supports the development of coatings that serve to decrease oxygen and moisture absorption. Moreover, the multiple peak values at δ 74–73 ppm indicate the presence of C2-C4 carbons within the sugar ring structure. Resonances observed around 79 and 101 ppm correlate to the glycosidic link between carbons C-1 and C-4. The results obtained at δ 60 ~ 85 ppm were interpreted as pyranose, supporting the FTIR evaluations (Benmebarek et al., 2024; Pasupathi et al., 2024).

#### 2.14.4 SEM and EDX analysis

**Figure 14:**
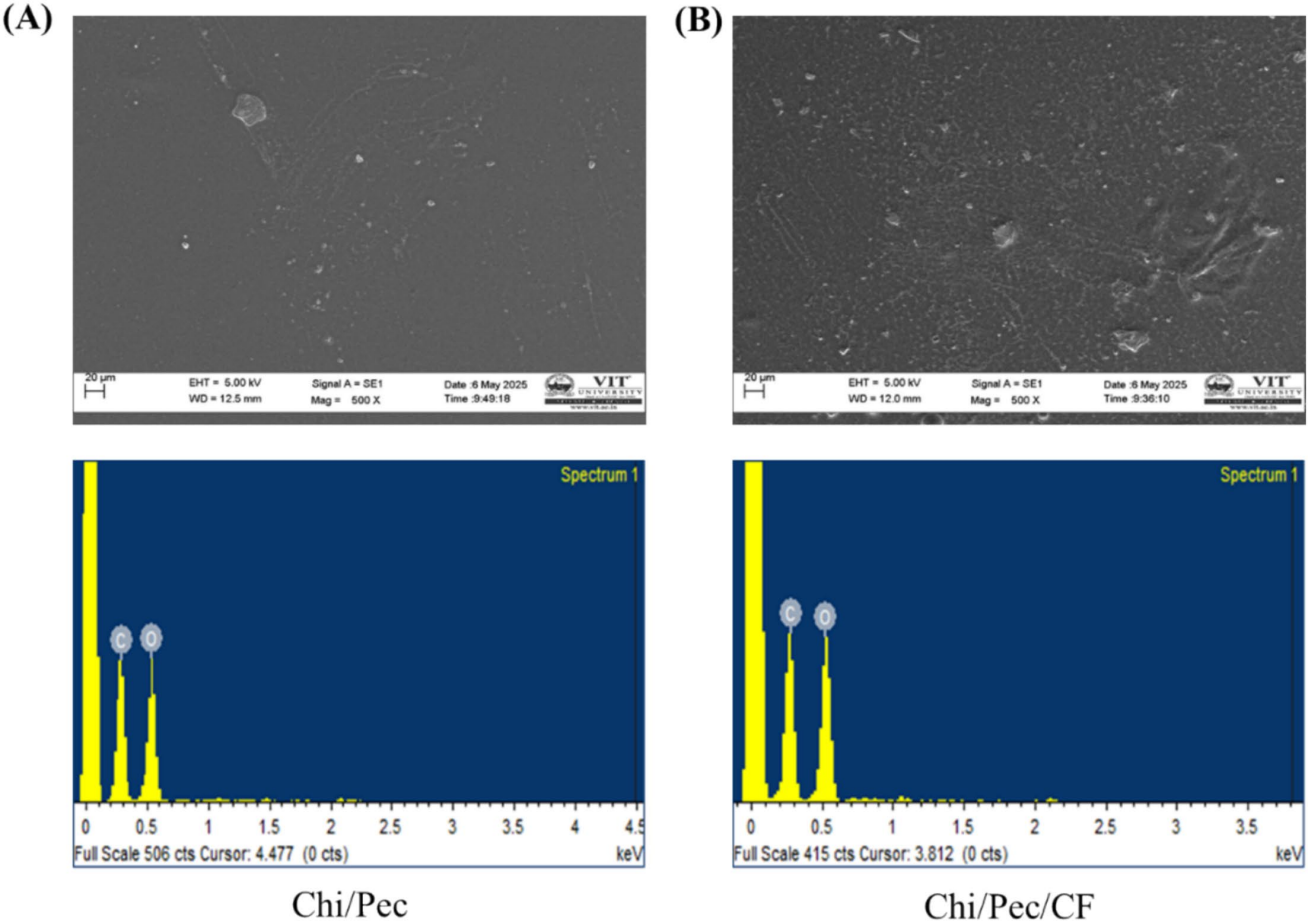
SEM/EDX micrograph of edible films **(A)** Chi/Pec film and **(B)** Chi/Pec/CF film.

SEM is utilized to assess the surface and interior structure of edible films. Figure 14 illustrates the morphological structure of Chi/Pec and Chi/Pec/CF films to elucidate the intricate microstructure of each film sample, enhancing structural understanding regarding the bioactive substances and polymer (Shivangi et al., 2021). The surface of each film shows that there are neither cracks nor bubbles, indicating a cohesive matrix. Micrographs of both films exhibit flawless, uniform, and abundant microstructures without breaks, demonstrating superior compatibility, excellent adherence, and reliability between them. The control film shows a flatter and smoother appearance, while the addition of the active fraction results in an opaquer appearance, suggesting a greater interaction between the fraction and polymer along with a rough appearance. Upon the addition of CF into the film samples, the film’s exterior exhibits a rather smooth surface with numerous pores, indicating the partial dispersion of bioactive compounds from the fraction on the film surface. A similar result was observed, where changes occurred in the morphology of films after the addition of *piper betle* leaf extract in pectin/chitosan composite films for the preservation of purple eggplants (Nguyen et al., 2022). Other studies reported on loquat leaf extract with cornstarch and *pequi* peel extract with chitosan films (Breda et al., 2017). EDX provides qualitative and quantitative data regarding the composition of elements and dispersion among the films (El Habbasha et al., 2025). To confirm the presence of elements in both control and blended films, the atomic weights of carbon (49.31 and 49.35) and oxygen (50.69 and 50.65) are relatively similar in both the control and blended films. This helps evaluate the efficiency of extracts in the film matrix. The interactions between the polymer and bioactive fraction enhance the moisture barrier strength of the active film in SEM (Kumar et al., 2021).

### 2.15 Thermal stability of edible films

**Figure 15:**
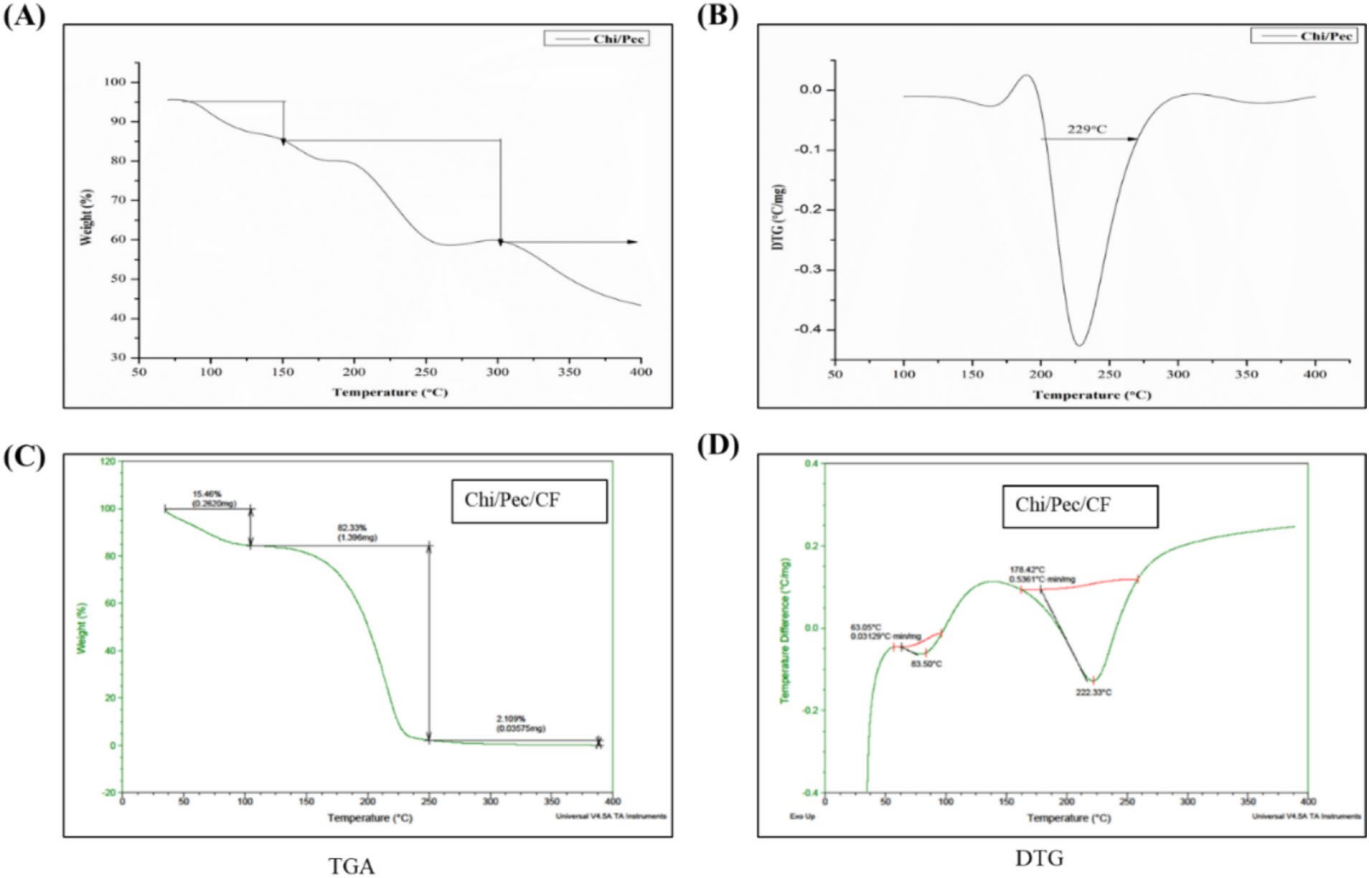
Thermogravimetric analysis of edible films using TGA and DTG curves **(A,B)** Chi/Pec films **(C,D)** Chi/Pec/CF films.

The thermal strength of Chi/Pec and Chi/Pec/CF films was analyzed using thermogravimetric properties under nitrogen gas conditions, as shown in Figure 15. Both films exhibited weight loss on the thermogravimetric analysis (TGA) curves, whereas the derivative thermogravimetry (DTG) curves represent maximal decomposition conditions during degradation and thermal decomposition. It shows that the polymer with an active fraction demonstrates three consecutive weight loss steps. The initial step was observed at 50–100 °C, with a weight loss of about 15.46%, due to moisture loss; the second stage occurred at 100–250 °C, with a weight loss of about 82.33%, indicating the degradation of biopolymers and glycerol; and the final step was evidenced at 250–400 °C, with a weight loss of about 2.109%, based on the oxidation of the polymer and bioactive components. In the DTG analysis, the composite films exhibited maximal decomposition at 222.33 °C, which is higher than that of the other films, and similar findings were reported for chitosan with silver nanoparticle films (Rodrigues et al., 2020). It was found that the film matrix showed higher values than the control film, indicating that they are thermally stable and increase the stability of the Chi/Pec/CF matrix.

### 2.16 Determination of antioxidant properties of edible films by DPPH assay

**Figure 16:**
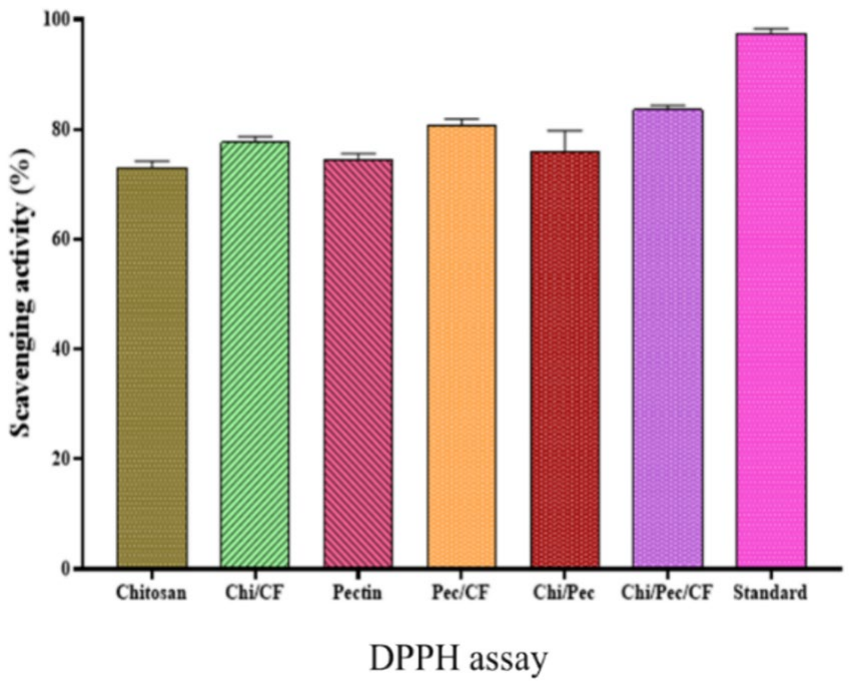
Antioxidant properties of edible films by DPPH assay.

The production of reactive substances by oxidative processes is a primary factor responsible for deterioration in the food industry. The incorporation of naturally occurring antioxidants into packaging for food components represents an innovative approach to preserving food in packages (Priyadarshi et al., 2018). In the current study, DPPH was used to estimate the potential antioxidant capabilities, and one of the primary antioxidant techniques is shown in Figure 16 and Table 8, which compare Chitosan, Chi/CF, Pectin, Pectin/CF, Chi/Pec, Chi/Pec/CF, and standard ascorbic acid. In the DPPH assay, the Chi/Pec/CF film showed an increased antioxidant capacity of 83.55 ± 0.64 compared to the control Chi/Pec film of 75.98 ± 3.08, followed by Pec/CF and Chi/CF, with the differences among the samples being statistically significant (*p* < 0.05). The incorporation of the bioactive fraction into the films significantly enhances their DPPH scavenging properties. Similar studies have also reported on edible films for DPPH, which indicate functional hydroxyl groups. This implies a correlation between scavenging and solution absorbance, which aids in the calculation of levels of antioxidants using this equation (Wang et al., 2016; Lei et al., 2019). A similar study was conducted with previous *L. sativum* extracts that showed greater scavenging activity for the film supplemented with LSE3 (Salem et al., 2021).

**Table 8:** Antioxidant activities of edible films by DPPH assay.

Samples	DPPH assay
Chitosan control	72.97 ± 1.07
Chi/CF	77.61 ± 0.90
Pectin control	74.46 ± 0.89
Pec/CF	80.77 ± 0.88
Chi/Pec control	75.98 ± 3.08
Chi/pec/CF	83.55 ± 0.64
Standard (ascorbic acid)	97.43 ± 0.72

The values are expressed as mean ± SD (*n* = 3). Statistical analysis utilized one-way ANOVA followed by Tukey’s *post-hoc* test.

### 2.17 Antimicrobial activity of edible films

**Figure 17:**
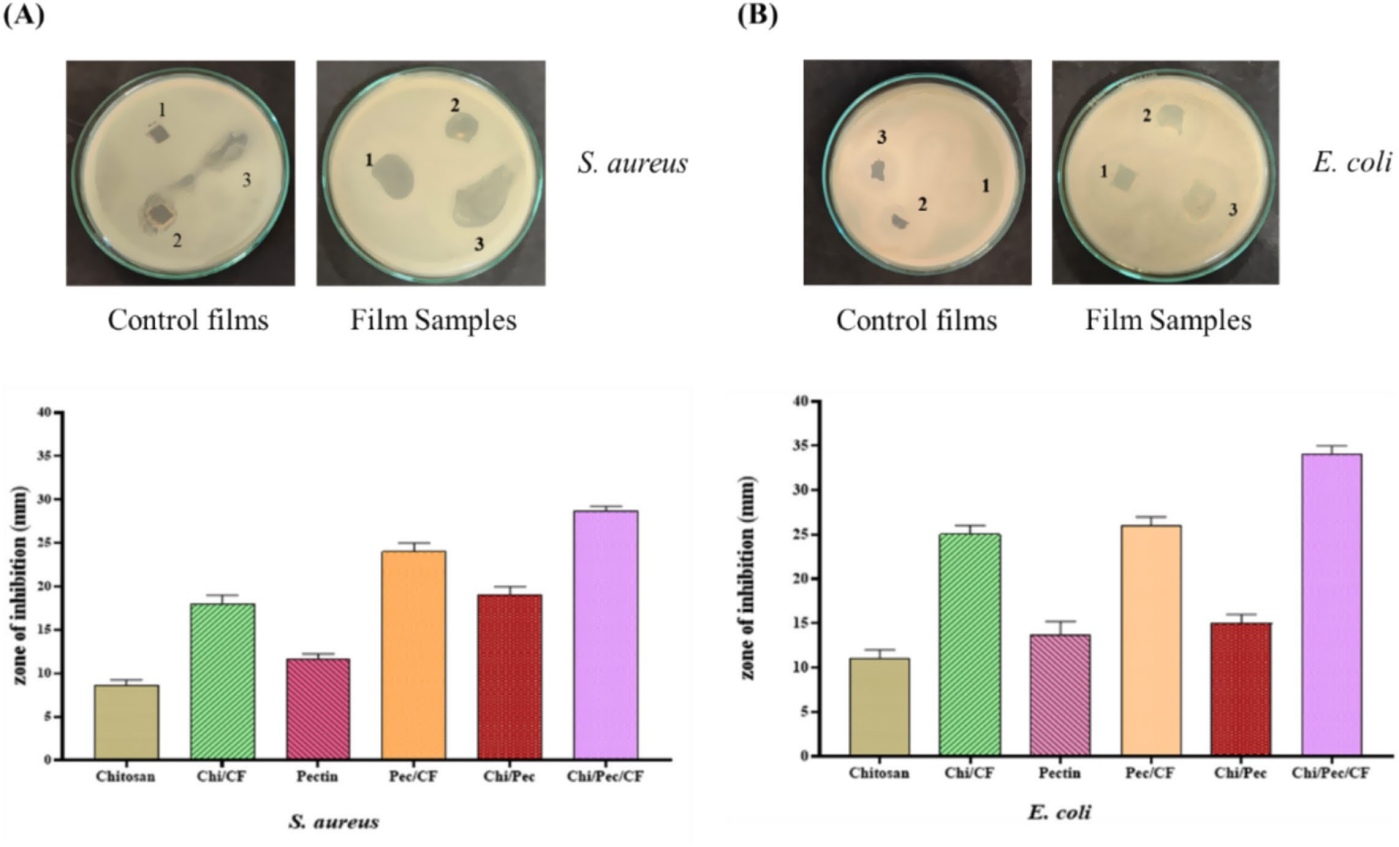
Antimicrobial efficacy of edible films **(A)** *S. aureus* **(B)** *E. coli.*

Foodborne microorganisms can significantly minimize the quality of foods and induce different illnesses during food preservation; because of this, food packaging films are crucial for their antimicrobial properties (Subramani and Manian, 2024). The antimicrobial effectiveness of edible films was evaluated against two foodborne organisms, *S. aureus* and *E. coli.* Representative antimicrobial plates and corresponding quantitative results are shown in Figure 17 and Table 9. Among the tested films, the Chi/Pec/CF film showed the highest inhibition zones in *S. aureus* and *E. coli* of 28 mm and 34 mm, whereas the control Chi/Pec film showed 19 mm and 15 mm, respectively. The results suggest that the inclusion of active fractions with bioactive substances enhances the antimicrobial potency of the film. Notably, Gram-positive bacterial strains were more sensitive to the films than Gram-negative bacterial strains. This difference can be ascribed to structural variations in the bacterial cell wall; Gram-negative bacterial strains possess a thicker exterior membrane composed of lipopolysaccharides, lipoproteins, and phospholipids, while Gram-positive bacterial strains have a peptidoglycan-dominant cell wall that is more permeable (Pu et al., 2024). Previous reports indicated that the addition of *Pistacia terebinthus*-derived extracts enhanced the antimicrobial activity (Kaya et al., 2018). Furthermore, similar studies have reported that pectin and blended films increase the efficacy of films in the mulberry leaf extract against foodborne pathogens such as *Pseudomonas aeruginosa* and *Bacillus cereus* (Shivangi et al., 2021).

**Table 9:** Antimicrobial activity of chitosan and Chi/CF films, pectin and Pec/CF films and Chi/Pec and Chi/Pec/CF films against food borne pathogens.

Microorganisms	Zone of inhibition (mm in dia)					
	Chitosan	Chi/CF	Pectin	Pec/CF	Chi/Pec	Chi/Pec/CF
*S. aureus*	8.66 ± 0.47	18 ± 0.81	11.66 ± 0.47	24 ± 0.81	19 ± 0.81	28 ± 0.47
*E. coli*	11 ± 0.81	25 ± 0.81	13.66 ± 1.24	26 ± 0.81	15 ± 0.91	34 ± 0.71

### 2.18 Biodegradability analysis of edible films

**Figure 18:**
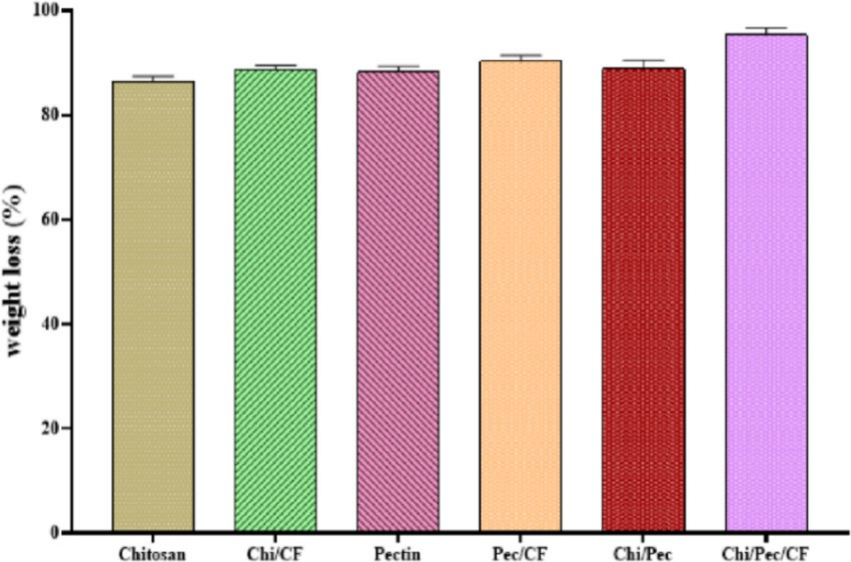
Weight loss of edible films after 20 days buried in soil.

The process of degradation involves the activity of organisms that disintegrate the edible films into smaller particles and subsequently break them down into simpler components like water, carbon dioxide, and biomass. In this study, biodegradability was assessed by simulating natural soil conditions where indigenous microflora facilitate degradation, as shown in Figure 18. Films buried in humid environmental conditions (Supplementary Figure S3) became brittle, swollen, and easily breakable. Weight loss was observed after 20 days of testing, resulting from microbial enzymatic activity and the dissolution of soluble substances in the films due to soil moisture. The Chi/Pec/CF film indicated 95.35%, followed by Pec/CF (90.23%), Chi/Pec film (88.37%), Chi/CF (88.65%), Pectin (88.27%), and Chitosan (86.33%), respectively. This confirms the effective biodegradable properties of the films. The result aligns with prior studies indicating that pectin-based films exhibit 95.13% degradability (Weldearegay et al., 2024). Furthermore, similar reports have shown that the addition of *Pistacia terebinth* extract leads to increased degradation of chitosan-based films in soil (Kaya et al., 2018).

### 2.19 Preservation of fresh cut apple slices using edible films

**Figure 19:**
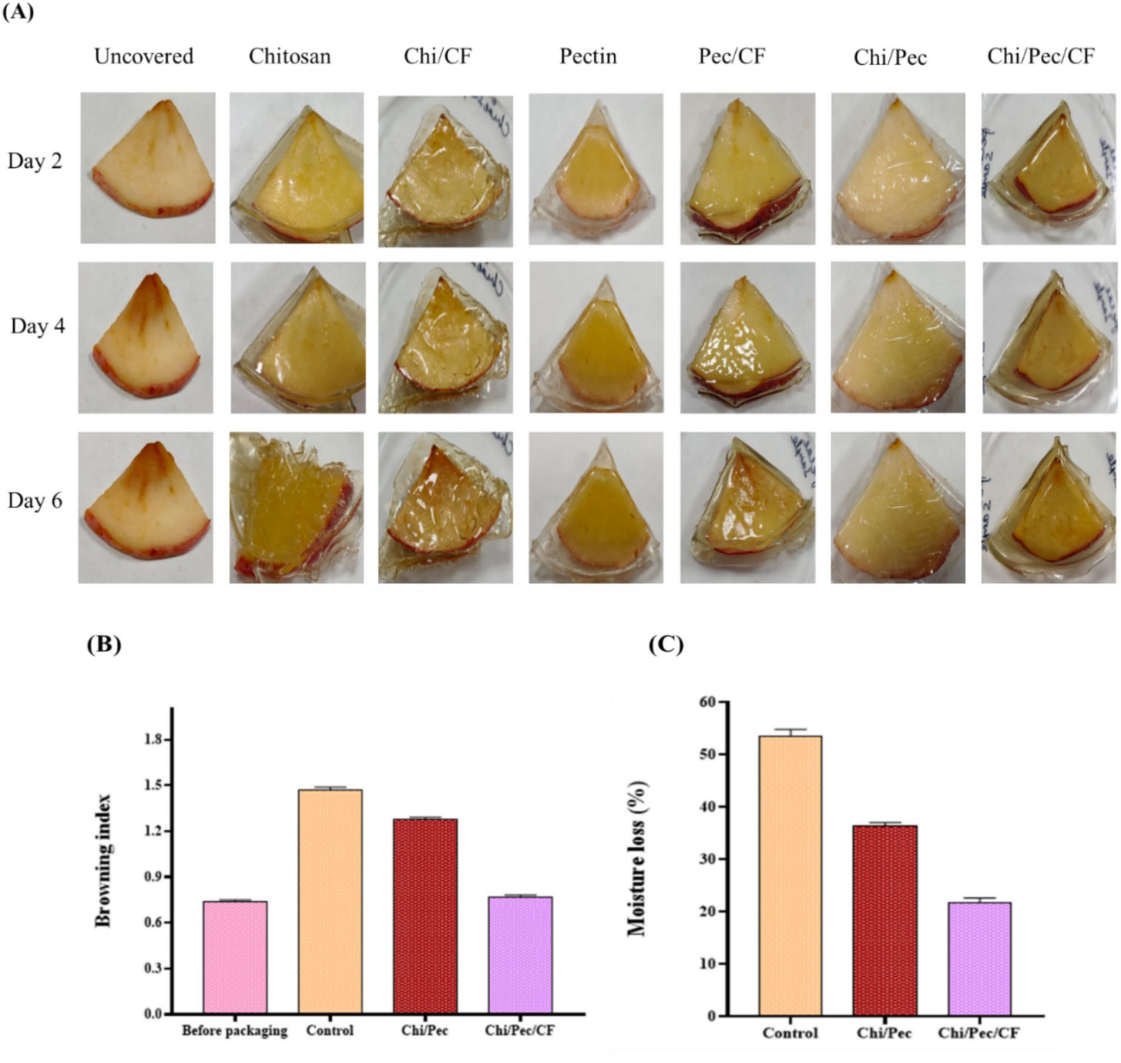
**(A)** Visual appearance of edible films on fresh cut apple slices stored at 4 °C for 6 days **(B,C)** effect of Chi/Pec and Chi/Pec/CF films on the browning level and moisture loss of freshly cut apple pieces.

The effectiveness of the films in food preservation and packaging was evaluated through weight loss and browning index analyses in Figure 19A. Fresh-cut apple pieces enclosed in these films generally undergo moisture loss due to the packaging materials’ reduced humidity relative to the ambient air (Vimala Bharathi et al., 2020; Subramani and Manian, 2024). This indicates that the films exhibit more water vapor permeability, which leads to higher moisture loss, whereas lower water vapor permeability leads to less moisture loss. Since the apple pieces were uncovered, it is evident that the exposed fruit slices exhibited more weight loss in our scenario. Similar results were reported in a study that utilized Agar–silver nanoparticle film for apple packaging (Gudadhe et al., 2014). As shown in Figures 19B,C, apple slices coated with Chi/Pec/CF film retained their natural color, whereas uncovered fruits exhibited a brown hue. The browning index was significantly higher in untreated fruits compared to those coated with composite films. Therefore, treated fruits were preserved at a similar level to the fresh-cut apples, demonstrating that edible films effectively delayed browning. This effect can be attributed to reduced WVP and enhanced antioxidant capabilities of the films, particularly those containing chitosan. Similar trends were reported in fresh-cut apple pieces with chitosan-blended films (El Mouzahim et al., 2023; Subramani and Manian, 2024).

## 3 Discussion

A number of factors, including solvent, technique, interval, temperature, and sample–solvent proportion, impact the extraction yield and efficiency. A crucial first step in identifying bioactive compounds is evaluating the biological activity of plant extracts, which involves selecting suitable extraction methods and solvents (Dowlath et al., 2020). Ethanol is the best solvent to extract bioactive compounds from *Cardiospermum halicacabum* leaves, illustrating the importance of solvent polarity in increasing plant metabolite extraction. Antioxidants affect DPPH because of their ability to donate electrons. One of the most popular, quick, and affordable techniques for measuring the antioxidant capabilities of plant extracts is the DPPH free radical scavenging assay (Başyiğit et al., 2020). Compared with all assays, the ethanol extract demonstrated the highest scavenging activity and potential for food preservation and packaging. Furthermore, the antibacterial activity of the ethanol extract of *C. halicacabum* against test organisms may support its ethnomedical use as a treatment for infections, skin rashes, mouth sores, aches, and boils. The results obtained suggest the development of natural biomaterials for food product packaging, potentially helping to prolong the storage life of wrapped food commodities. Avoiding the use of synthetic preservatives in food processing has become increasingly popular recently. In response to growing concerns about food safety, natural antimicrobials have been increasingly developed to control foodborne and spoilage microorganisms (Ihegboro et al., 2020).

Though chromosomal aberrations and mitotic abnormalities were observed in the *Allium cepa* assay, the alterations were concentration-dependent and occurred under controlled experimental exposure conditions. The *A. cepa* model is broadly accepted as a preliminary plant-based cytogenetic screening system; however, the results acquired from this assay cannot be directly extrapolated to human toxicological responses. In the present study, the extract is intended for incorporation into edible film matrices, where the release of bioactive compounds is regulated and likely to limit direct cellular exposure compared with the *in vitro* assay conditions. Nonetheless, the reported genotoxic manifestations highlight the need for careful dose optimization and the establishment of clearly defined safety thresholds prior to practical food-contact applications. Consequently, comprehensive toxicological evaluations such as mammalian cell culture studies, *in vivo* safety assessments, and regulatory compliance analyses are needed to substantiate biosafety and validate the suitability of the extract for food packaging applications (Brand-Williams et al., 1995). Furthermore, quantitative estimation of identified constituents, such as total phenolic and flavonoid contents, could enhance the dependability of these results. Phenolic and flavonoid compounds are of particular importance because of their reported antimicrobial and antioxidant activities (Venkatesan and Muniyan, 2024).

It is important to emphasize that phytocomponent identification was conducted solely by comparison of mass spectra with reference library databases and was not confirmed using authentic standards, retention index validation, or compound isolation. In addition, detailed similarity index values were not available in the exported GC–MS dataset. Accordingly, the reported compound assignments should be considered tentative. The molecular docking analyses were conducted using the proposed chemical structures under the assumption of correct identification. Therefore, the docking results should be interpreted as preliminary and hypothesis-generating. Further analytical verification, involving structural confirmation and quantitative assessment of individual constituents, is necessary to establish the specific bioactive components responsible for the observed antibacterial activity.

The *in vitro* antibacterial findings were further supported through molecular docking analysis, which provided theoretical insights into potential ligand–target interactions. The identified compounds demonstrated favorable binding affinities toward the selected target proteins, suggesting probable interactions at the molecular level. However, molecular docking represents a predictive computational approach and does not confirm actual enzyme inhibition or antimicrobial mechanisms.

Variations among experimental and computational outcomes highlight the inherent limitations of docking studies, as they cannot fully account for protein flexibility, solvent effects, cellular complexity, metabolism, or bioavailability. Therefore, the docking results should be interpreted as hypothesis-generating tools that assist in prioritizing bioactive compounds and proposing potential mechanisms. Experimental validation through enzyme inhibition assays or mechanistic studies is required to substantiate the predicted interactions (Trinh et al., 2025).

Moreover, the ethanol extract of *C. halicacabum* shows promise for additional pharmacological applications. Column chromatography yielded several fractions that were eluted and estimated for antimicrobial activity, and the most active fraction exhibited greater growth inhibition. This active fraction was further applied in the formulation of an edible film for food preservation applications. The developed edible film, made from a composite of chitosan and pectin and enriched with fractions of *C. halicacabum*, acts as a novel natural substance for food preservation and packaging. These bioactive fraction-based films were characterized and effectively extended the shelf life of apple slices, demonstrating potential as an alternative to artificial preservatives. Although the apple slices exposed to the extract indicated reduced browning and loss of moisture during refrigerated storage (4 °C), microbial enumeration was not conducted in the current study. Therefore, conclusions regarding microbiological shelf-life extension remain preliminary. Future investigations should incorporate total viable count, yeast and mold analysis, and extended storage duration to comprehensively validate the preservation efficiency of the extract under real food system conditions. This research offers valuable insights that could assist food producers and researchers in developing new, safe, and effective preservatives.

### 3.1 Limitations of the study

Overall, our outcomes imply that the identified hit components possess multipurpose characteristics, including antioxidant capacity, antimicrobial efficacy, and favorable binding interactions with microbial targets. The present study highlights the possibility of antioxidant and antibacterial features of *C. halicacabum* extracts. Although substantial antibacterial activity of the ethanolic extract was demonstrated using the agar well diffusion method, a limitation of this study is that minimum inhibitory concentration (MIC), minimum bactericidal concentration (MBC), and time-kill kinetics were not determined. Furthermore, microbial load (log CFU/g) was not quantified during the apple storage study, which would provide direct evidence of microbial reduction in the food matrix. While the edible films demonstrated preliminary protective effects, the concentrations of extract used are relatively high, and potential impacts on sensory properties were not evaluated. Future studies will aim to analyze MIC and MBC values for a more quantitative evaluation of antimicrobial efficiency. Additional research is also needed to assess cytotoxicity, conduct *in vivo* animal studies, and evaluate sensory effects in real food matrices. These investigations will provide a comprehensive understanding of the practical applications and safety of *C. halicacabum* extracts as natural food preservatives.

## 4 Conclusion

This study assessed the phytochemical composition of *C. halicacabum* using various solvent extracts, as well as its antioxidant, antibacterial, and cytotoxic properties. The results demonstrated that the ethanolic extract showed the highest antioxidant potential, mostly due to its abundant concentration of antioxidant substances. The ethanolic extract exhibited extensive antimicrobial activity against both bacterial strains. Cytotoxicity was evaluated using the *A. cepa* test, which demonstrated efficacy as a preliminary bioassay for assessing the genotoxic and toxic characteristics of plant-derived compounds prior to prospective environmental or biomedical application. Furthermore, the bioactive fraction of the leaf of *Cardiospermum halicacabum* ethanolic extract combined with chitosan and pectin to create an edible film for the first time. The structural details and spectroscopy investigations validated the compatibility and integrating capacity of the edible films. The bio-composite films exhibiting the highest biological capabilities include Chi/Pec/CF (83%) for the DPPH assay, whereas Chi/Pec films (75%) affirm their appropriateness for food preservation and packaging applications. Moreover, Chi/Pec/CF surpassed the other films in preserving the moisture content and freshness of the apple pieces. In conclusion, edible film composed of polymers and fractions appears to be a promising substance for the food packaging industry. Further understanding of the additional characteristics of biological inquiry is essential, which includes examining the chemical interactions among the food product and packaging substance, as well as purifying the compounds from the extracts.

## Data Availability

The original contributions presented in the study are included in the article/Supplementary material, further inquiries can be directed to the corresponding author.

## Footnotes

1
https://pubchem.ncbi.nlm.nih.gov

2
https://www.3ds.com/products/biovia/discovery-studio

3
https://plasma-gate.weizmann.ac.il/Grace/

4
https://pubchem.ncbi.nlm.nih.gov/
