# Supplementary material for: Antimicrobial evaluation of Cardiospermum halicacabum L. leaf fractions and its usage in active bioactive film formation for shelf-life enhancement in fresh-cut fruits
Source: Front Microbiol. 2026 Apr 1;17:1812196. doi: 10.3389/fmicb.2026.1812196 (PMC13079059; doi:10.3389/fmicb.2026.1812196)
Supplement: Supplementary file 1 [file Data_Sheet_1.docx]

**Antimicrobial evaluation of *Cardiospermum halicacabum* L. leaf fractions and its usage in active bioactive film formation for shelf-life enhancement in fresh-cut fruits**

**1. SUPPLEMENTARY FIGURES**

**
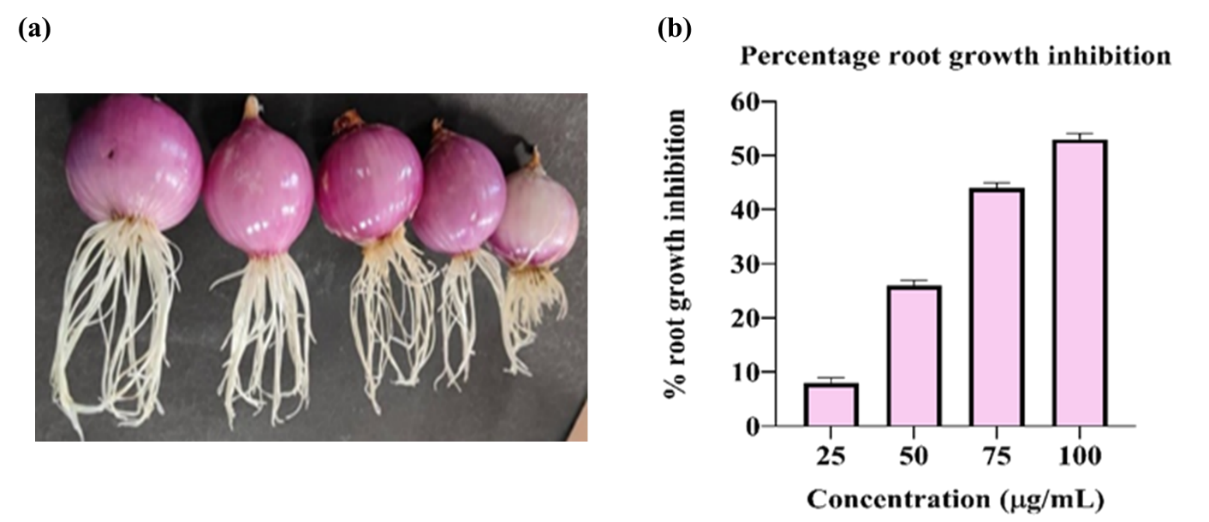
**

**SUPPLEMENTARY FIGURE S1** (a) *A. cepa* treated to different concentrations of the ethanolic extract of *C.halicacabum* leaves indicated the following macroscopic effects and (A) negative control, (B) 25, (C) 50, (D) 75 and (E) 100 µg/mL. (b) After 48 hours of exposure to various concentrations of the ethanolic extract of *C.halicacabum* leaves and the control, the percentage of root growth inhibition in *A. cepa*.


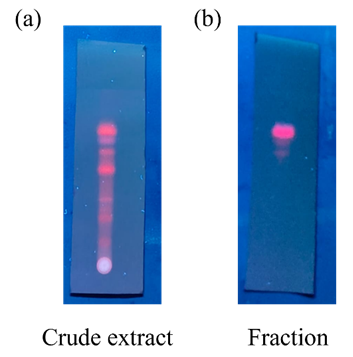


**SUPPLEMENTARY FIGURE S2** TLC profiling of *C. halicacabum* ethanolic extract visualized underneath ultraviolet light at 365nm (a) Crude extract (b) Fraction


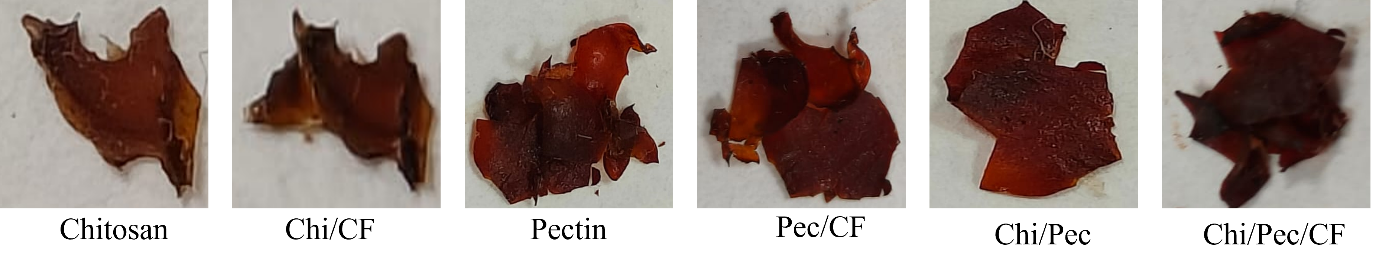


**SUPPLEMENTARY FIGURE S3** Degradation of entire films sample after 20 days of being buried in the soil

**2. SUPPLEMENTARY TABLES**

**SUPPLEMENTARY TABLE S1** Percentage yield of the extracts made from *C. halicacabum* plant using various solvents

| **Medicinal plant** | **Solvents** | **The initial weight of the sample (g)** | **Weight of dried extract (g)** | **Yield of extraction (%)** |
| --- | --- | --- | --- | --- |
| *C. halicacabum* | Petroleum ether | 25 | 3.2 | 16.2 |
|  | Chloroform |  | 4.3 | 21.5 |
|  | Ethanol |  | 4.8 | 24.2 |

**SUPPLEMENTARY TABLE S2** TLC profiling of crude extract and fraction

| **Crude extract**  **(Toluene: Ethyl acetate 4:1 ratio)** | **Fraction**  **(Toluene: Ethyl acetate 4:1 ratio)** |
| --- | --- |
| 0.21 | 0.81 |
| 0.31 | - |
| 0.57 | - |
| 0.68 | - |
| 0.74 | - |
| 0.78 | - |

**SUPPLEMENTARY TABLE S3** Antimicrobial efficacy (zone of inhibition) determined by fractions against food-borne pathogens and values represented as mean ± standard deviation

| **Fractions** | **Zone of inhibition (mm) diameter** | |
| --- | --- | --- |
|  | ***S. aureus*** | ***E. coli*** |
| 1 | - | - |
| 2 | 13.66 ± 2.05 | - |
| 3 | 14 ± 1.63 | - |
| 4 | 15.66 ± 2.06 | 11± 0.81 |
| 5 | 17.33 ± 3.09 | 12 ± 0.82 |
| Tetracycline (Positive control) | 27.66 ± 3.08 | 37 ± 1.41 |
| Negative control | - | - |

**SUPPLEMENTARY TABLE S4** Phytochemical components were identified in the ethanolic extract of *C. halicacabum* using gas chromatography-mass spectrometry (GC-MS)

| **S.no** | **Compound name** | **Molecular formula** | **Molecular weight (g/mol)** | **Retention time** | **Area%** | **Structure** | **Biological activities** |
| --- | --- | --- | --- | --- | --- | --- | --- |
| 1. | 4'-Pentylbicyclohexyl-4-carboxamide | [C_18_H_33_NO](https://pubchem.ncbi.nlm.nih.gov/#query=C18H33NO) | 279.5 | 5.330 | 0.443 | 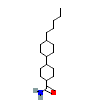 | Plant metabolite (Syatkin et al., 2024) |
| 2 | 2-Formyl-9-[beta-d-ribofuranosyl] hypoxanthine | [C_11_H_12_N_4_O_6_](https://pubchem.ncbi.nlm.nih.gov/#query=C11H12N4O6) | 296.24 | 5.450 | 0.450 | 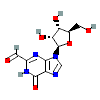 | Silver recovery (Parimi et al., 2024) |
| 3 | 2-Hydroxymethyl-9-[beta-d-ribofuranosyl] hypoxanthine | [C_11_H_14_N_4_O_6_](https://pubchem.ncbi.nlm.nih.gov/#query=C11H12N4O6) | 298.25 | 5.575 | 0.746 | 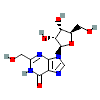 | New compound |
| 4 | 9-[3,4-dihydroxy-5-(hydroxymethyl) oxolan-2-yl]-2-ethenyl-3H-purin-6-one | [C_12_H_14_N_4_O_5_](https://pubchem.ncbi.nlm.nih.gov/#query=C12H14N4O5) | 294.26 | 6.895 | 0.375 | 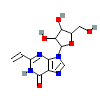 | New compound |
| 5 | 3-O-Methylhexopyranose | [C_7_H_14_O_6_](https://pubchem.ncbi.nlm.nih.gov/#query=C7H14O6) | 194.18 | 11.597 | 0.334 | 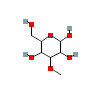 | New compound |
| 6 | N-(5-Chloro-2-hydroxyphenyl) dodecanamide | [C_18_H_28_ClNO_2_](https://pubchem.ncbi.nlm.nih.gov/#query=C18H28ClNO2) | 325.9 | 11.837 | 0.925 | 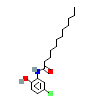 | Plant metabolite (Linfield et al., 1983) |
| 7 | (2,3,4-triacetyloxy-5-cyano-5-oxopentyl) acetate | [C_14_H_17_NO_9_](https://pubchem.ncbi.nlm.nih.gov/#query=C14H17NO9) | 343.29 | 11.992 | 1.673 | 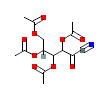 | New compound |
| 8 | Sucrose | [C_12_H_22_O_11_](https://pubchem.ncbi.nlm.nih.gov/#query=C12H22O11) | 342.30 | 12.137 | 0.783 | 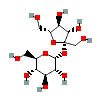 | Food additives (Chang et al., 2025) |
| 9 | Pentadecanoic Acid | [C_15_H_30_O_2_](https://pubchem.ncbi.nlm.nih.gov/#query=C15H30O2) | 242.40 | 13.853 | 0.945 | 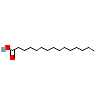 | Flavoring agent (Han et al., 2025) |
| 10 | D-Mannoheptulose-13C7 | [C_7_H_14_O_7_](https://pubchem.ncbi.nlm.nih.gov/#query=C7H14O7) | 217.13 | 14.263 | 0.820 | 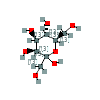 | New compound |
| 11 | 4-O-Methylhexopyranose | [C_7_H_14_O_6_](https://pubchem.ncbi.nlm.nih.gov/#query=C7H14O6) | 194.18 | 14.358 | 0.362 | 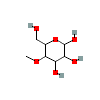 | Plant metabolite (Effah and Clavijo McCormick, 2024) |
| 12 | Oleic Acid | [C_18_H_34_O_2_](https://pubchem.ncbi.nlm.nih.gov/#query=C18H34O2) | 282.5 | 14.468 | 0.495 | 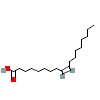 | Food additives (Chen et al., 2025) |
| 13 | 9-Octadecenal | C_18_H_34_O | 266.5 | 15.234 | 8.080 | 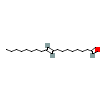 | Volatile compound and flavoring agents (Dias et al., 2025) |
| 14 | 2-Octylcyclopropene-1-heptanol | [C_18_H_34_O](https://pubchem.ncbi.nlm.nih.gov/#query=C18H34O) | 266.5 | 15.554 | 6.527 | 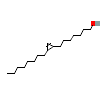 | New compound |
| 15 | 2-Trifluoroacetoxytridecane | [C_15_H_27_F_3_O_2_](https://pubchem.ncbi.nlm.nih.gov/#query=C15H27F3O2) | 296.37 | 15.949 | 11.498 | 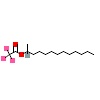 | Computational studies (Surendirakumar et al., 2024) |
| 16 | Oxalic acid, isohexyl pentyl ester | C_13_H_24_O_4_ | 244.33 | 16.099 | 5.586 | 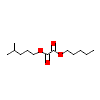 | New compound |
| 17 | Oxalic acid, cyclobutyl nonyl ester | [C_15_H_26_O_4_](https://pubchem.ncbi.nlm.nih.gov/#query=C15H26O4) | 270.36 | 16.504 | 30.402 | 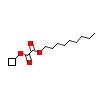 | New compound |
| 18 | 1-Octadecyne | C_18_H_34_ | 250.5 | 16.874 | 6.184 | 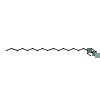 | Plant metabolite (Bachhar et al., 2024) |
| 19 | Palmitic Acid | [C_16_H_32_O_2_](https://pubchem.ncbi.nlm.nih.gov/#query=C16H32O2) | 256.42 | 17.185 | 15.037 | 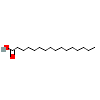 | Controlled lipid homeostasis (Li et al., 2025) |
| 20 | cis-9,10-Epoxyoctadecan-1-ol | [C_18_H_36_O_2_](https://pubchem.ncbi.nlm.nih.gov/#query=C18H36O2) | 284.5 | 17.280 | 4.677 | 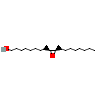 | New compound |
| 21 | 3-(Prop-2-enoyloxy) dodecane | C_15_H_28_O_2_ | 240.38 | 17.560 | 0.564 | 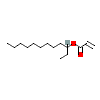 | New compound |
| 22 | 7,11-Hexadecadienal | C_16_H_28_O | 236.39 | 17.725 | 0.330 | 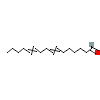 | Associated antioxidant activity (Prabakaran et al., 2017) |
| 23 | Laminitol | [C_7_H_14_O_6_](https://pubchem.ncbi.nlm.nih.gov/#query=C7H14O6) | 194.18 | 17.750 | 0.305 | 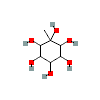 | Associated antibacterial activity (Lo et al., 2019) |
| 24 | glycero-galacto-Heptose | C_7_H_14_O_7_ | 210.18 | 17.900 | 0.600 | 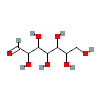 | Plant metabolite (Chingizova et al., 2025) |
| 25 | Methyl beta-D-mannofuranoside | [C_7_H_14_O_6_](https://pubchem.ncbi.nlm.nih.gov/#query=C7H14O6) | 194.18 | 18.990 | 0.827 | 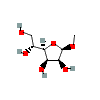 | New compound |
| 26 | 13-Tetradecen-11-yn-1-ol | [C_14_H_24_O](https://pubchem.ncbi.nlm.nih.gov/#query=C14H24O) | 208.34 | 20.346 | 0.417 | 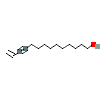 | Associated antioxidant activity (Nithiyanandam and Evan Prince, 2023) |
| 27 | 3-O- Methylglucose | [C_7_H_14_O_6_](https://pubchem.ncbi.nlm.nih.gov/#query=C7H14O6) | 194.18 | 26.683 | 0.615 | 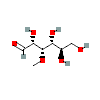 | Associated antioxidant activity (Hassan et al., 2025) |

**SUPPLEMENTARY TABLE S5** Analysis of hit components by toxicity

| **Compounds/Control** |  | **Hepatotoxicity** | **Carcinogenicity** | **Immunotoxicity** | **Cytotoxicity** | **Mutagenicity** |
| --- | --- | --- | --- | --- | --- | --- |
| Hit 1 | Prediction | Inactive | Inactive | Inactive | Inactive | Inactive |
|  | Probability | 0.79 | 0.54 | 0.97 | 0.77 | 0.90 |
| Hit 2 | Prediction | Inactive | Inactive | Inactive | Inactive | Inactive |
|  | Probability | 0.65 | 0.72 | 0.99 | 0.85 | 0.79 |
| Hit 3 | Prediction | Inactive | Inactive | Inactive | Inactive | Inactive |
|  | Probability | 0.65 | 0.73 | 0.99 | 0.90 | 0.85 |
| Control 1 | Prediction | Inactive | Inactive | Inactive | Inactive | Inactive |
|  | Probability | 0.67 | 0.62 | 0.99 | 0.62 | 0.65 |
| Control 2 | Prediction | Inactive | Active | Active | Inactive | Inactive |
|  | Probability | 0.54 | 0.60 | 0.95 | 0.56 | 0.81 |

**3. SUPPLEMENTARY REFERENCES**

Bachhar, V., Joshi, V., Gangal, A., Duseja, M., and Shukla, R. K. (2024). Identification of Bioactive Phytoconstituents, Nutritional Composition and Antioxidant Activity of Calyptocarpus vialis. *Appl Biochem Biotechnol* 196, 1921–1947. doi: 10.1007/s12010-023-04640-5

Chang, G., Liu, J., Feng, B., Chen, J., Huang, Q., Liu, G., et al. (2025). Establishment and Methodology Validation of Latex-Enhanced Turbidimetric Immunoassay for Dextran Determination in Sugar. *Sugar Tech* 27, 1742–1751. doi: 10.1007/s12355-025-01612-3

Chen, H., Zhao, Q., Chi, G., Chen, K., Wang, Z., and Kan, J. (2025). Effects of the oleic acid-rich glycerolipids and thermal-mechanical treatment on the functionality of wheat gluten: Multi-spectroscopy and molecular simulation analysis. *Food Chem* 484, 144472. doi: 10.1016/j.foodchem.2025.144472

Chingizova, E. A., Novikova, O. D., Portnyagina, O. Yu., and Aminin, D. L. (2025). Components of Bacterial Cell Walls as Targets for Searching for New Antibacterial Compounds: Methods of Study. *Mol Biol* 59, 293–319. doi: 10.1134/S0026893325700013

Dias, S. R., Bressani, A. P. P., Batista, N. N., Dias, D. R., and Schwan, R. F. (2025). Increasing the quality and complexity of pulped coffee fermentation with Lactiplantibacillus plantarum and selected yeasts. *Eur Food Res Technol* 251, 283–297. doi: 10.1007/s00217-024-04640-7

Effah, E., and Clavijo McCormick, A. (2024). Invasive Plants’ Root Extracts Display Stronger Allelopathic Activity on the Germination and Seedling Growth of a New Zealand Native Species than Extracts of Another Native Plant or Conspecifics. *J Chem Ecol* 50, 1086–1097. doi: 10.1007/s10886-024-01550-6

Han, H., Kuai, Z., Yao, Z., Lei, X., Shi, H., Li, J., et al. (2025). Exploring the influence of sex and age on the quality and flavor of Huai goat longissimus thoracis et lumborum through transcriptomic and metabolomic analyses. *BMC Genomics* 26, 684. doi: 10.1186/s12864-025-11857-7

Hassan, M., Khamis, G., Zorkany, H. E., and Alexeree, S. (2025). Innovative approaches to enhancing tamarind seed germination and phytochemical production through laser irradiation: implications for photodynamic therapy. *Lasers Med Sci* 40, 290. doi: 10.1007/s10103-025-04527-3

Li, L., Ren, X., Gao, X., Zhao, Y., Ma, Y., Gong, J., et al. (2025). Correction: Multiomics analysis revealed the regulatory role of Chenodeoxycholic acid in fatty acid metabolism and lipid homeostasis. *Lipids Health Dis* 24, 318. doi: 10.1186/s12944-025-02752-x

Linfield, W. M., Micich, T. J., Montville, T. J., Simon, J. R., Murray, E. B., and Bistline, R. G. (1983). Antibacterially active substituted anilides of carboxylic and sulfonic acids. *J. Med. Chem.* 26, 1741–1746. doi: 10.1021/jm00366a016

Lo, H.-J., Chang, Y.-K., Ananthan, B., Lih, Y.-H., Liu, K.-S., and Yan, T.-H. (2019). Total Synthesis of (+)-Lycoricidine and Conduramine B-1, ent-C-1, C-4, D-1, ent-F-1, and ent-F-4, and Formal Synthesis of (-)-Laminitol: a C2-Symmetric Chiral-Pool-Based Flexible Strategy. *J Org Chem* 84, 10065–10075. doi: 10.1021/acs.joc.9b01221

Nithiyanandam, S., and Evan Prince, S. (2023). Caesalpinia bonducella mitigates oxidative damage by paracetamol intoxication in the kidney and intestine via modulating pro/anti-inflammatory and apoptotic signaling: an In vivo mechanistic insight. *3 Biotech* 13, 176. doi: 10.1007/s13205-023-03601-3

Parimi, M. R., Gantala, D. D., Deepala, N. A., Bikkina, S. S. Y., Reddy, J. M. K., and Vangalapati, M. (2024). Optimization of Bromelain extraction from green papaya (Carica papaya) peel waste and its application in the recovery of silver from waste of X-ray photographic films. *Environ Sci Pollut Res*. doi: 10.1007/s11356-024-35435-8

Prabakaran, S., Ramu, L., Veerappan, S., Pemiah, B., and Kannappan, N. (2017). Effect of different solvents on volatile and non-volatile constituents of red bell pepper (Capsicum annuum L.) and their in vitro antioxidant activity. *Food Measure* 11, 1531–1541. doi: 10.1007/s11694-017-9532-3

Surendirakumar, K., Devi, W. S., and Vaithilingam, S. (2024). Exploring the in silico studies of the endophyte fungus Phoma herbarum against mur enzymes of Staphylococcus aureus – a computational approach. *Vegetos* 37, 2470–2480. doi: 10.1007/s42535-023-00738-7

Syatkin, S. P., Blagonravov, M. L., Hilal, A., Sungrapova, K. Yu., Sokuev, R. I., Korzun, I. A., et al. (2024). Influence of Some Heterocyclic, Cyclic, and Nitrogen-Containing Compounds on Oxidative Deamination of Polyamines in a Cell-Free Test System. *Bull Exp Biol Med* 177, 307–312. doi: 10.1007/s10517-024-06179-9
